# Unravelling Novel Phytochemicals and Anticholinesterase Activity in Irish *Cladonia portentosa*

**DOI:** 10.3390/molecules28104145

**Published:** 2023-05-17

**Authors:** Shipra Nagar, Maria Pigott, Wirginia Kukula-Koch, Helen Sheridan

**Affiliations:** 1 NatPro Centre, School of Pharmacy and Pharmaceutical Sciences, Trinity College Dublin, Dublin 02, D02 PN40 Dublin, Ireland; mpigott@tcd.ie; 2 Department of Pharmacognosy, Medical University of Lublin, 1 Chodzki Street, 20-093 Lublin, Poland; virginia.kukula@gmail.com

**Keywords:** usnic acid, olivetolic acid, 4-*O*-methylolivetolcarboxylic acid, perlatolic acid, LC-MS, fragmentation mechanism, placodiolic acid, pseudoplacodiolic acid

## Abstract

Acetylcholinesterase inhibitors remain the mainstay of symptomatic treatment for Alzheimer’s disease. The natural world is rich in acetylcholinesterase inhibitory molecules, and research efforts to identify novel leads is ongoing. *Cladonia portentosa*, commonly known as reindeer lichen, is an abundant lichen species found in Irish Boglands. The methanol extract of Irish *C. portentosa* was identified as an acetylcholinesterase inhibitory lead using qualitative TLC-bioautography in a screening program. To identify the active components, the extract was deconvoluted using a successive extraction process with hexane, ethyl acetate and methanol to isolate the active fraction. The hexane extract demonstrated the highest inhibitory activity and was selected for further phytochemical investigations. Olivetolic acid, 4-*O*-methylolivetolcarboxylic acid, perlatolic acid and usnic acid were isolated and characterized using ESI-MS and two-dimensional NMR techniques. LC-MS analysis also determined the presence of the additional usnic acid derivatives, placodiolic and pseudoplacodiolic acids. Assays of the isolated components confirmed that the observed anticholinesterase activity of *C. portentosa* can be attributed to usnic acid (25% inhibition at 125 µM) and perlatolic acid (20% inhibition at 250 µM), which were both reported inhibitors. This is the first report of isolation of olivetolic and 4-*O*-methylolivetolcarboxylic acids and the identification of placodiolic and pseudoplacodiolic acids from *C. portentosa*.

## 1. Introduction

A lichen is a self-supporting and stable combination of two symbiotic partners: a mycobiont and a photobiont. The mycobiont, which is a fungal partner, provides water, physical protection and mineral resources, while the photobiont can be an algal partner or cyanobacteria [1]. The photobiont performs photosynthesis, using water and carbon dioxide to produce carbohydrates. Lichens produce primary and secondary metabolites [2]. Secondary metabolites, known as lichenic acids, are mainly produced by the fungal partner, such as an *Ascomycete* or *Basidiomycete* [3]. Lichenic acids demonstrate antibiotic, antifungal, anti-inflammatory, antiviral, analgesic, antipyretic and antioxidant activities [4,5]. Therefore, they may have potential in the pharmaceutical industry. The production of secondary metabolites by lichens can be affected by many environmental factors, such as physical distribution, the pH of the substrate and vegetation dynamics, especially the climatic parameters and rock substrates. For instance, fumarprotocetraric acid is produced in high yield on alkaline substrates, whereas the relationship is reversed for the triterpene metabolite zeorin. Similarly, a dry environment offering hot temperatures and low rainfall favors the production of the depside atranorin [6,7]. Furthermore, high levels of ammonia in the atmosphere directly affect the nitrogen content in lichens and influence their metabolic pathways [8,9]. Lichens can grow on many substrates, such as trees, rocks and soil, and species can tolerate the extreme climates of freezing polar regions or dry and hot deserts [10]. The same lichen species can have distinct metabolites and thus unique chemical fingerprints when collected at different geographical locations, from different environments and during different seasons [11]. Lichens can survive in harsh conditions such as extremely low temperatures, scarce precipitation and scant nutrient availability due to their ability to maintain a metabolic resting condition for months [12]. Additionally, for ecological reasons, they may produce metabolites exhibiting antimicrobial, phytotoxic and cytotoxic activities to protect themselves against opportunistic microscopic and macroscopic predators present in the surrounding environment [13,14].

*Cladonia portentosa*, a member of the genus *Cladoniaceae*, also known as reindeer lichen, is widespread in the Irish boglands [15]. This lichen belongs to one of the fruticose groups and is light green to grey-white in color and squamulose in shape [2,16]. An *Ascomycete* fungus is the fungal partner for *Cladonia* species. It bears a fruiting body, called an apothecium, that produces spores asexually to proliferate [17]. The fungus finds an appropriate algal partner (usually *Asterochloris* sp. in the case of *Cladonia* spp.) and associates with it to form a stable symbiotic relationship in the form of lichen [18]. The secondary metabolites reported for *C. portentosa* include a dibenzofuran, usnic acid and a depside, perlatolic acid [19,20]. Both have been reported to have potent cytotoxicity [21], while usnic acid also exhibits antifungal potential against *Phytophthora infestans* and *Ustilago maydis* [19]. Usnic acid is one of the most ubiquitous lichenic acids, and it exists in its different enantiomeric forms in different species. For instance, *Usnea* and *Ramalina* spp. are known for (+)-usnic acid, while *Alectoria* sp. has (-)-usnic acid. Interestingly, both enantiomers have been reported in *Cladonia* spp. *C. arbuscula* and *C. mitis* contains (+)-usnic acid, while *C. uncialis*, *C. portentosa*, *C. stellaris* and *C. foliacea* are known for containing (-)-usnic acid [22,23].

The Unlocking Nature’s Pharmacy from Bogland Species (UNPBS) Project (grant number DOJProject209825) is focused on investigating the potential of using the flora of the Irish boglands as a source of novel plant-derived therapeutics. In phase 1, which involved screening of plant and lichen species collected from Irish bogs, *Cladonia portentosa* was identified as a potential acetylcholinesterase (AChE) inhibitory lead using a qualitative TLC-bioautography assay. The clinical uses of AChE inhibitors include treatment of Alzheimer’s disease, glaucoma and myasthenia gravis [24]. Alzheimer’s disease is a progressive, neurodegenerative disease responsible for the majority of cases of dementia [25]. Its pathogenesis is not fully understood, and there is no cure [26]. AChE inhibitors are the mainstay of symptomatic treatment for Alzheimer’s disease. Levels of acetylcholine and other neurotransmitters are reduced in the disease, and increasing the level of cholinergic transmission through inhibition of acetylcholine hydrolysis is beneficial, particularly for mild to moderately severe cases of Alzheimer’s dementia. The natural world is a rich source of AChE inhibitory molecules [27,28] which continues to furnish novel drug entities with the potential to meet the clinical need for new therapeutics for diseases such as Alzheimer’s. Lichens are a known source of AChE inhibitory leads [29]. While the inhibitory activity of lichen extracts may be attributed to extensively studied metabolites such as usnic acid, bioguided extract selection and deconvolution has also resulted in the isolation of new inhibitory leads such as the AChE inhibitor biruloquinone which was extracted from *Cladonia macilenta* [30]. The activity of *C. portentosa* was studied using an HPTLC-bioautography and colorimetric microplate assays of the methanol extract and extracts of stratified polarity obtained using a successive extraction process with hexane, ethyl acetate and methanol. Guided by the bioassays, the hexane extract was selected for fractionation using column chromatography to isolate and characterise the components and delineate the metabolites responsible for the anticholinesterase activity of *C. portentosa*.

## 2. Results

*C. portentosa* lichen material was cleaned, dried and milled, and extracts were prepared using the Soxhlet extraction method. The prepared extracts and yields are given in Table 1. The successive extraction process provided simplified extracts for further isolation and characterisation steps.

### 2.1. HPLC Analysis

**Figure 1:**
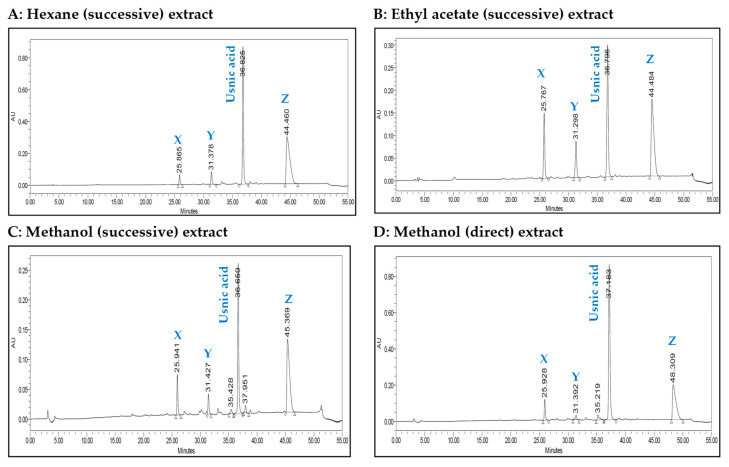
HPLC chromatograms of successive and direct extracts of *C. portentosa*.

The HPLC chromatograms of the four extracts are shown in Figure 1. The four chromatograms contain four major peaks at retention times of 25.8 min, 31.3 min, 36.8 min and 44.4 min. Usnic and perlatolic acids have been reported in *C. portentosa* [19], and usnic acid was available as a commercial standard. The peak eluting time at 36.8 min was identified for usnic acid compared with the authentic standard, while the remaining three unknown peaks were labelled as X, Y and Z.

### 2.2. LC-MS Analysis

**Figure 2:**
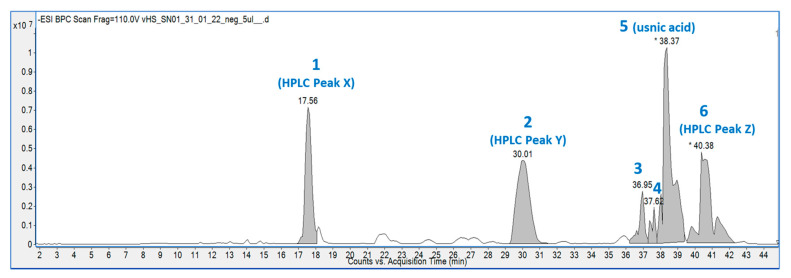
The total ion chromatogram of the hexane extract of *C. portentosa* in the negative ion mode. The peak numbers denote 6 compounds corresponding to Table 2. Peaks 1, 2 and 6 are the same compounds as X, Y and Z shown in the HPLC chromatograms in Figure 1. (* denotes manual peak integration).

**Figure 3:**
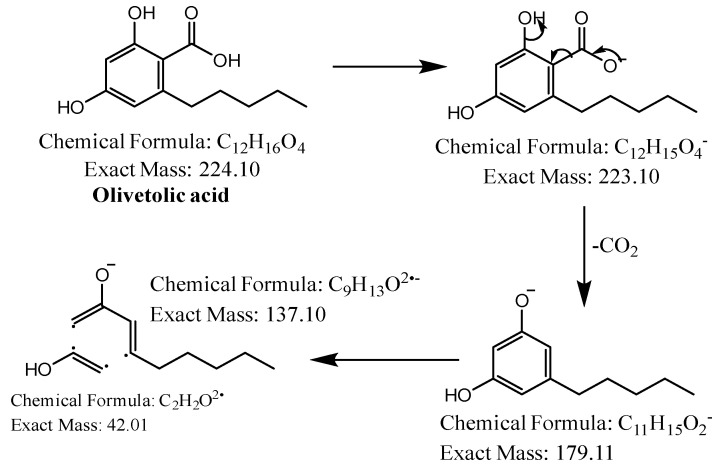
Proposed fragmentation of Olivetolic acid eluted at 17.6 min in the negative ion mode corresponding to the MS/MS spectrum (Table 2, Supplementary Figure S1). The pseudomolecular ion was produced as [M-H]^−^ at *m*/*z* 233.1 Da, which may break down to a fragment at *m*/*z* 205.1 Da corresponding to [M-H_2_O-H]^−^ (fragment not shown in Figure 3), along with other fragments at *m*/*z* 179.1 Da and 137.1 Da.

**Figure 4:**
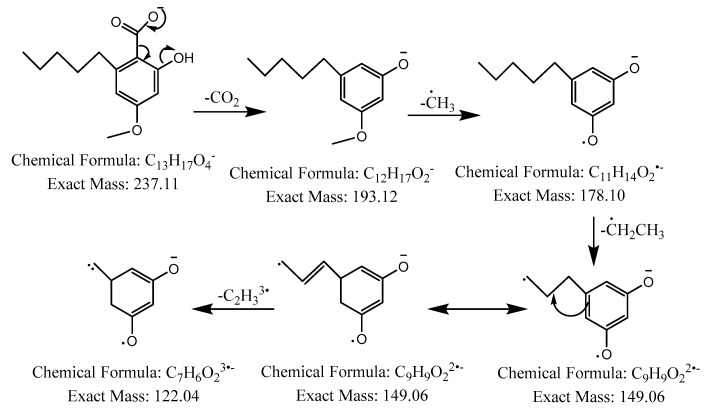
Proposed fragmentation of 4-*O*-methylolivetolcarboxylic acid eluted at 29.9 min in the negative ion mode, corresponding to the MS/MS spectrum (Table 2, Supplementary Figure S2). The pseudomolecular ion was produced as [M-H]^−^ at *m*/*z* 237.1 Da, which may undergo decarboxylation to produce a fragment at *m*/*z* 193.1 Da, followed by stepwise removal of the aliphatic chain to generate fragments at *m*/*z* 178.1 Da, 149.1 Da and 122.0 Da.

**Figure 5:**
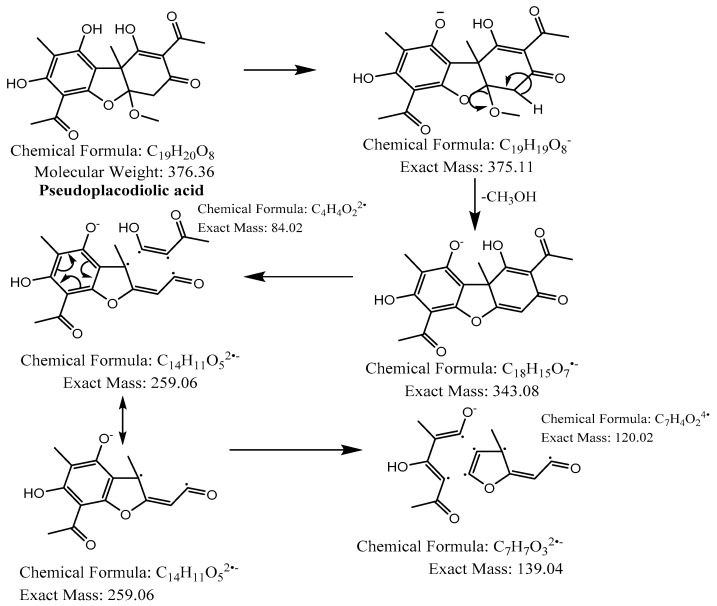
Proposed fragmentation of Pseudoplacodiolic acid eluted at 36.9 min or 37.6 min in the negative ion mode, corresponding to the MS/MS spectra (Table 2, Supplementary Figures S3 and S4). The pseudomolecular ion was produced as [M-H]^−^ at *m*/*z* 375.1 Da, which lost a methanol molecule and generated a daughter fragment at *m*/*z* 343.1 Da. The [M-H]^−^ ion may undergo decarboxylation in parallel to produce a fragment at *m*/*z* 299.1 Da (fragment not shown in Figure 5). The daughter ion at *m*/*z* 343.1 Da further breaks down to give fragments at *m*/*z* 259.1 Da and 139.0 Da.

**Figure 6:**
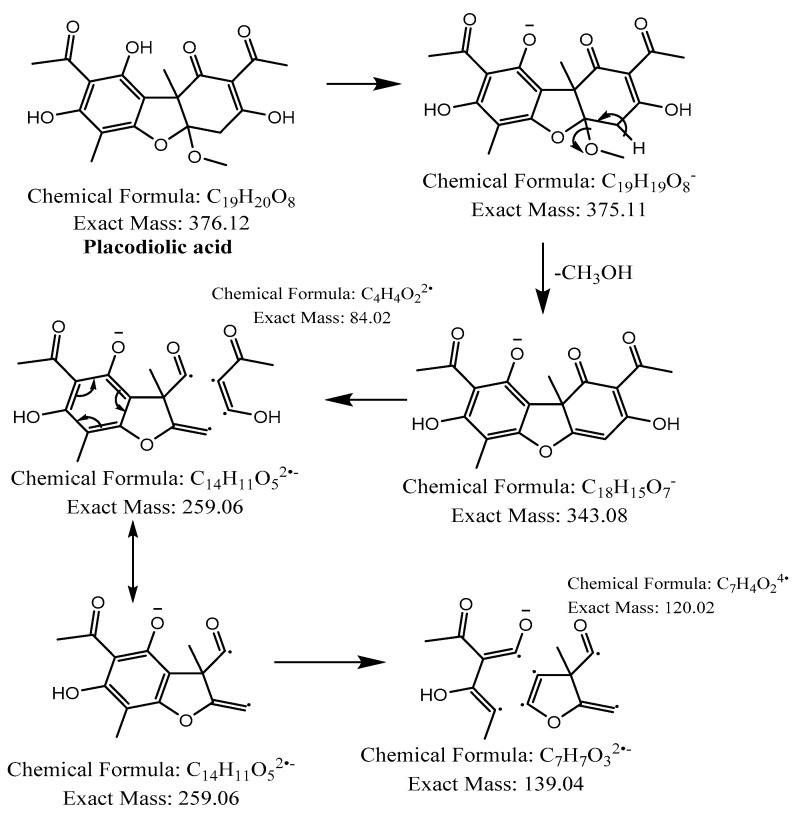
Proposed fragmentation of Placodiolic acid eluted at 36.9 min or 37.6 min in the negative ion mode, corresponding to the MS/MS spectra (Table 2, Supplementary Figures S3 and S4). The pseudomolecular ion was produced as [M-H]^−^ at *m*/*z* 375.1 Da, which lost a methanol molecule and generated a daughter fragment at *m*/*z* 343.1 Da. The [M-H]^−^ ion may undergo decarboxylation in parallel to produce a fragment at *m*/*z* 299.1 Da (fragment not shown in Figure 6). The daughter ion at *m*/*z* 343.1 Da further breaks down to give fragments at *m*/*z* 259.1 Da and 139.0 Da.

**Figure 7:**
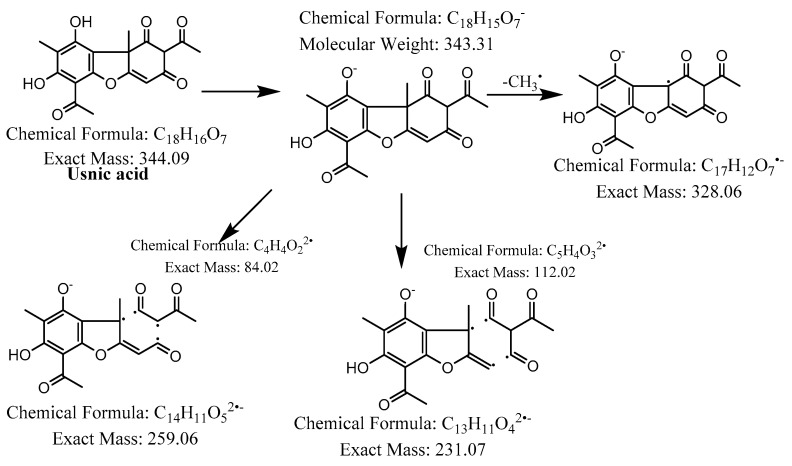
Proposed fragmentation of usnic acid eluted at 38.2 min in the negative ion mode, corresponding to the MS/MS spectra (Table 2, Supplementary Figure S5). The pseudomolecular ion was produced as [M-H]^−^ at *m*/*z* 343.3 Da, which followed different degradation paths in parallel. It may lose a methyl radical and generate a daughter fragment at *m*/*z* 328.1 Da. Alternatively, the [M-H]^−^ ion may be cleaved across the benzene ring of dibenzofuran to give fragments at *m*/*z* 259.1 Da and 231.1 Da.

**Figure 8:**
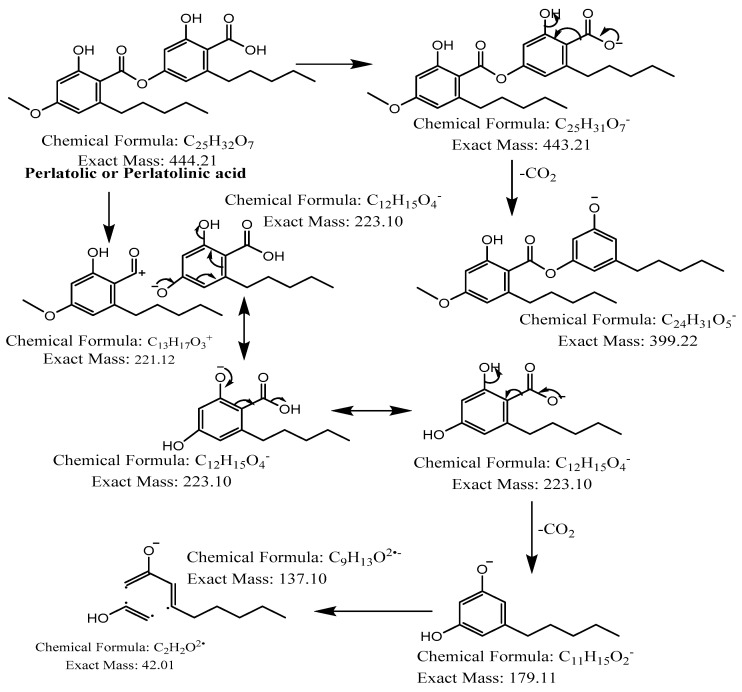
Proposed fragmentation of Perlatolic or Perlatolinic acid eluted at 40.3 min in the negative ion mode, corresponding to the MS/MS spectra (Table 2, Supplementary Figure S6). The pseudomolecular ion was produced as [M-H]^−^ at *m*/*z* 443.2 Da, which may undergo decarboxylation to give a fragment at *m*/*z* 399.2 Da. The parent ion [M] may undergo heterolytic cleavage of the ester bond of the depside to generate daughter ions of olivetolic acid at *m*/*z* 223.1 Da, which may release a water molecule to give a fragment [M-H_2_O-H]^−^ at *m*/*z* 205.1 Da (fragment not shown in Figure 8) or undergo decarboxylation and further break down into fragments with *m*/*z* 179.1 and 137.1 Da, respectively.

The hexane extract was submitted for chemical fingerprinting using liquid chromatography–mass spectrometry (LC–MS), and the spectra obtained in the negative ion mode in the total ion chromatogram were interpreted based on their fragmentation patterns (Figure 2, Figure 3, Figure 4, Figure 5, Figure 6, Figure 7 and Figure 8), retention times and the literature data.

In the high-resolution mass spectrometry-based study, six compounds were tentatively identified: olivetolic acid (**1**, HPLC peak X), 4-*O*-methylolivetolcarboxylic acid (**2**, HPLC peak Y), placodiolic acid (**3**), pseudoplacodiolic acid (**4**), usnic acid (**5**) and perlatolic or perlatonilic acid (**6**, HPLC peak Z). The spectrometric data obtained and used for identification of the compounds is presented in Table 2, and the fragmentation pathways of the tentatively identified compounds are presented in Figure 2, Figure 3, Figure 4, Figure 5, Figure 6, Figure 7 and Figure 8 and supplementary Figures S1–S6.

### 2.3. Isolation and Characterisation of Components

**Figure 9:**
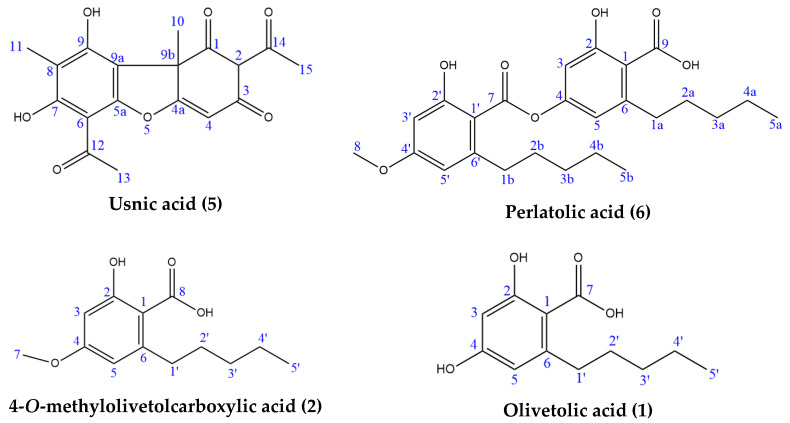
Lichenic acids isolated from *C. portentosa*.

Column chromatography of the hexane extract (300 mg) was performed using silica gel to isolate and confirm the identity of the components predicted by the LC–MS data analysis. Four lichenic acids were isolated as the major components: usnic (55 mg, **5**), perlatolic (38.2 mg, **6**), olivetolic (4.2 mg, **1**) and 4-*O*-methylolivetolcarboxylic (0.7 mg, **2**) acids (Figure 9). Usnic acid was further purified through recrystallization, and the other three acids were purified using preparative TLC. The yields of lichenic acids fractionated from the hexane extract, especially olivetolic and 4-*O*-methylolivetolcarboxylic acids, were low. Therefore, the methanol extract, which was obtained through direct extraction with a higher yield and has a similar HPLC chemical profile (Table 1, Figure 1), was also subjected to fractionation by column chromatography to obtain higher amounts of lichenic acids for further structural investigations. The four components were subjected to ESI-MS analysis to furnish exact molecular weight, and one-dimensional (^1^H NMR and ^13^C NMR) and two-dimensional (HSQC and HMBC) NMR were performed to characterise their chemical structures (Supplementary Figures S7–S14, Tables S1–S4). Table 3 shows the NMR assignment of the chemical structures of the four molecules and comparison to the literature.

Usnic acid exhibits chirality, and its specific rotation was measured along with commercial (+)-usnic acid. The specific rotation of the isolated compound was found to be −380 deg (c = 0.1% in chloroform, 25 °C), while the (+)-enantiomer was +495 deg. The enantiomeric excess in the isolated usnic acid was found to be 76.7%.

### 2.4. Probing the Anticholinesterase Activity of Cladonia portentosa

**Figure 10:**
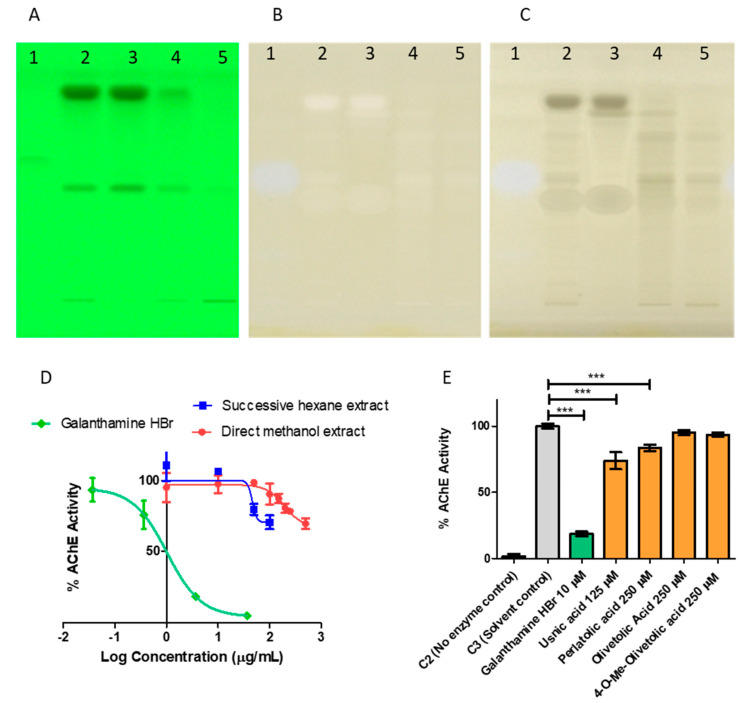
Probing of the acetylcholinesterase (AChE) inhibitory activity of *Cladonia portentosa* extracts by HPTLC-bioautography and colorimetric microplate assay. HPTLC-bioautography (Panels **A**–**C**). Panel **A**: Developed plate under 254 nm before derivatization. Panel **B**: Plate derivatized for the detection of AChE inhibitory activities under white light in remission. Panel **C**: Plate derivatized for the detection of AChE inhibitory activities under white light in transmission. Lane 1—Galanthamine HBr, 5 mM, 5 μL; Lane 2—direct methanol extract of *C. portentosa,* 1.66 mg/mL, 5 μL; Lane 3—successive hexane extract of *C. portentosa,* 1.66 mg/mL, 5 μL; Lane 4—successive ethyl acetate extract of *C. portentosa,* 1.66 mg/mL, 5 μL; Lane 5—successive methanol extract of *C. portentosa,* 1.66 mg/mL, 5 μL. A white spot with a blue hue was obtained for the known inhibitor galanthamine HBr under white light in remission and transmission. For the *Cladonia* extracts, white zones, appearing white in remission, appeared black in transmission. Colorimetric microplate assay (Panels **D**,**E**). Panel **D**: Enzyme inhibition curves of the direct methanol extract and the successive hexane extract of *C. portentosa* and the known AChE inhibitor Galanthamine HBr, IC_50_ = 1.024 µg/mL or 2.71 µM. Data plotted as mean ± SD. Panel **E**: AChE inhibition by lichenic acids isolated from *C. portentosa*. Statistical analysis by One-way ANOVA with Dunnett post test. *** indicates *p*-value < 0.0001. The data are represented as the mean ± SD of experimental triplicates.

High-throughput screening using manual TLC-bioautography on aluminum-backed TLC plates revealed that *C. portentosa* methanol extract possesses AChE inhibitory potential. AChE inhibitors yield white zones of inhibition on a yellow background when plate derivatisation is performed. The enzyme hydrolyses the substrate acetylthiocholine iodide (ATCI) to thiocholine on the plate which reacts with 5,5′-dithio-bis(2-nitrobenzoic acid) (DTNB) (Ellman’s reagent), cleaving it to form yellow 2-nitro-5-thiobenzoate [31]. The screening method based on the Ellman reaction [32] was transferred to a semi-automated HPTLC system. White spots on the yellow background appeared for the *Cladonia portentosa* extracts. However, the *Cladonia* spots behaved differently in visualisation compared to the spot for galanthamine, the positive control for AChE inhibition. Galanthamine yielded a blue-white zone that was visible in both white light in remission (lit from above) and white light in transmission (lit from beneath). However, for *Cladonia* samples, the spots appeared white in remission, but lacked the blue hue observed for galanthamine, and were black in transmission (Figure 10, Panels A, B and C). The possibility that this divergent behaviour was due to false positives prompted us to probe the AChE inhibitory activity in a colorimetric microplate assay. Both the direct methanol extract and the successive hexane extract were found to have some weak inhibitory effects on the enzyme, reducing the observed in vitro activity by approximately 30% at a test concentration of 500 μg/mL for the direct methanol extract and at a test concentration of 100 μg/mL for the hexane extract, which were the highest concentrations examined for both. Galanthamine hydrobromide was used as a positive control for enzyme inhibition in the assay, and the IC_50_ value was determined as 1.024 µg/mL or 2.71 µM (Figure 10, Panel D). The concentration ranges used in the assay were limited by the aqueous solubility of the extracts, and it remained unclear whether greater efficacy could be achieved with an increased concentration of extract or extract component. Delineation of the contributing components in the more potent hexane extract was pursued.

**Table 3:** ^1^H (600 Hz) and ^13^C (150 Hz) NMR spectral data assignment for usnic acid, perlatolic, 4-O-methylolivetolcarboxylic and olivetolic acid; ^A^ DMSO-d6, ^B^ acetone-d6, ^C^ chloroform-d, ^D^ methanol-d4.

Position	Usnic Acid				Position	Perlatolic Acid				4-*O*-methylolivetolcarboxylic Acid				Olivetolic Acid			
	Isolated		Literature [33]			Isolated		Literature [34]		Isolated		Literature [34]		Isolated		Literature [34]	
	^δ^H ^A^	^δ^C ^A^	^δ^H ^A^	^δ^C ^A^		^δ^H ^A^	^δ^C ^A^	^δ^H ^A^	^δ^C ^B^	^δ^H ^C^	^δ^C ^C^	^δ^H ^C^	^δ^C ^C^	^δ^H ^A^	^δ^C ^A^	^δ^H ^D^	^δ^C ^B^
**1**	-	197.9	-	197.0	1	-	118.7	-	110.9	-	111.3	-	114.4	-	105.2	-	104.2
**2**	-	105.4	-	105.8	2-OH	-	156.9	-	165.1	-	164.9	-	165.2	10.00 (s)	163.5	-	167.2
**3**	-	191.4	-	190.9	3	6.59 (d)	106.8	6.57 (d)	109.2	6.33 (d)	98.9	6.35 (s)	99.0	6.12 (d)	100.5	6.14 (d)	101.6
**3-OH**	11.28 (s)		11.33 (s)	-	4	-	151.7	-	154.9	-	166.8	-	166.8	-	-	-	
**4**	6.31 (s)	98.3	6.31 (s)	98.8	4-OH	-	-	-	-	-	-	-	-	8.97 (s)	161.5	-	163.4
**4a**		179.3		178.8	5	6.50 (d)	113.0	6.48 (d)	116.5	6.35 (d)	103.4	6.35 (s)	103.5	6.16 (d)	109.9	6.19 (d)	111.5
**5**	-	-	-	-	6	-	143.7	-	149.3	-	149.5	-	149.9	-	147.1	-	149.8
**5a**		155.4	-	155.4	7	-	166.6	-	173.2	3.82 (s)	55.5	3.82 (s)	-	-	172.7	-	174.0
**6**	-	101.2	-	101.1	7-OH	-	-	-	-	-	-	-	-	11.81 (s)	-	-	-
**7**	-	156.7	-	157.0	8	3.74 (s)	55.2	3.73 (s)	55.8	-	-		-	-	-	-	-
**7-OH**	13.36 (s)	-	13.37 (s)	-	8-OH	-	-	-	-	11.70 (s)	-	-	-	-	-	-	-
**8**	-	107.2	-	107.0	9-OH	-	169.7	-	-	-	-		-	-	-	-	-
**9**		162.6		162.5	1′	-	106.8	-	105.0	2.91 (t)	36.8	2.93 (t)	36.8	2.74 (t)	35.2	2.88 (t)	37.0
**9-OH**	13.36 (s)		13.37(s)		2′	10.24 (s)	161.7	-	166.4	1.59 (m)	32.1	1.60 (m)	32.1	1.47 (m)	30.9	1.57 (m)	32.2
**9a**	-	104.9	-	105.1	3′	6.36 (d)	98.9	6.35 (d)	99.7	1.34 (m)	31.6	1.36 (m)	31.5	1.28 (m)	31.3	1.35 (m)	32.6
**9b**	-	58.7	-	58.2	4′	-	157.9	-	165.5	1.34 (m)	22.6	1.36 (m)	22.6	1.28 (m)	21.8	1.35 (m)	22.9
**10**	1.74 (s)	31.5	1.74 (s)	31.6	5′	6.36 (d)	111.6	6.35 (d)	111.5	0.89 (m)	14.2	0.91 (t)	14.2	0.85 (t)	13.9	0.91 (t)	14.1
**11**	2.00 (s)	7.6	2.00 (s)	7.6	6′	-	142.9	-	148.8	-	-	-	-	-	-	-	-
**12**	-	201.0	-	201.0	1a	2.63 (m)	33.3	2.58 (m)	36.8	-	-	-	-	-	-	-	-
**13**	2.67 (s)	31.1	2.67 (s)	31.1	2a	1.55 (m)	30.4	1.53 (m)	32.6	-	-	-	-	-	-	-	-
**14**	-	201.3	-	201.0	3a	1.28 (m)	31.1	1.27 (m)	32.3	-	-	-	-	-	-	-	-
**15**	2.59 (s)	27.8	2.59 (s)	28.3	4a	1.28 (m)	21.8	1.27 (m)	23.0	-	-	-	-	-	-	-	-
**-**	-	-	-	-	5a	0.85 (t)	13.8	0.83 (t)	14.5	-	-	-	-	-	-	-	-
**-**	-	-	-	-	1b	2.63 (m)	33.8	2.62 (m)	37.5	-	-	-	-	-	-	-	-
**-**	-	-	-	-	2b	1.55 (m)	30.8	1.53 (m)	32.8	-	-	-	-	-	-	-	-
**-**	-	-	-	-	3b	1.28 (m)	31.2	1.27 (m)	32.6	-	-	-	-	-	-	-	-
**-**	-	-	-	-	4b	1.28 (m)	21.9	1.27 (m)	23.2	-	-	-	-	-	-	-	-
**-**	-	-	-	-	5b	0.85 (m)	13.9	0.84 (t)	14.3	-	-	-	-	-	-	-	-

Four major lichenic acids were isolated and characterised from the *C. portentosa* hexane extract: **1**, **2**, **5** and **6**. The AChE inhibitory activity of the acids was assayed using a colorimetric microplate assay (Figure 10, Panel E). Among them, **5** and **6** were found to inhibit the enzyme. Due to poor solubility in the aqueous buffer assay medium, the maximum concentration of **5** that was tested was 125 μM, which inhibited the enzyme by approximately 25% compared to the solvent control. At a higher concentration of 250 μM, **6** provided 20% inhibition, while **1** and **2** did not inhibit the enzyme in vitro at concentrations of up to 250 μM. Both **5** and **6** have been reported in the literature as AChE inhibitors [35,36]. The AChE inhibitory activity of *Cladonia portentosa* extracts can be attributed to the presence of usnic acid and perlatolic acid.

## 3. Discussion

*C. portentosa* was extracted using methanol and successively extracted with hexane, ethyl acetate and methanol, resulting in four extracts (Table 1). The direct methanol extraction yielded twice the amount compared to the successive method, as it was able to extract both non-polar and polar metabolites from the lichen. With the successive approach, non-polar and semi-polar metabolites were extracted by using hexane and ethyl acetate prior to the successive methanol extraction. Following identification of the direct methanol extract as an AChE inhibitory lead, successive extractions were performed with the aim to targeting active metabolites. The hexane extract, which was most effective at inhibiting AChE, was selected for further phytochemical investigations. Four major peaks were detected in the HPLC chromatograms of the four *C. portentosa* extracts (Figure 1). One peak was determined to be **5** (36.8 min), while the remaining three unknown peaks were labelled as X (25.8 min), Y (31.3 min) and Z (44.4 min). The hexane extract was subjected to LC-ESI-MS measurements, which provided a chromatogram in the negative ion mode (Figure 2, Table 2) with six peaks, including four major peaks corresponding to the HPLC run. To ensure that compounds X, Y and Z corresponded to the major peaks at RT 17.6 min, 30.0 min and 40.38 min in the LC–MS chromatogram, the sample was run in HPLC using the LC–MS method, and the order and intensity of the peaks were matched. Compounds **5** and **6** were expected metabolites in *C. portentosa* [37], and two of the six peaks in the LC–MS chromatograms corroborated this conclusion. The remaining four peaks were identified based on fragmentation mechanisms interpreted from their tandem MS spectra and the literature.

The first peak at RT 17.6 min was identified as olivetolic acid (**1**, HPLC peak X)) with a pseudomolecular ion of [M-H]^−^ at *m*/*z* 233.1 Da (Figure 3). The second peak at RT 29.9 min was identified as 4-*O*-Me-olivetolcarboxylic acid (**2**, HPLC peak Y) with a pseudomolecular ion of [M-H]^−^ at *m*/*z* 237.1 Da (Figure 4). The third and fourth peaks at RT 36.9 min and 37.6 min had a low intensities and gave a [M-H]^−^ at *m*/*z* 375.1 Da, which corresponded to the molecular formulae C_19_H_20_O_8_ and C_18_H_16_O_9_. We found in the literature [34] that the following four lichenic acids biosynthesised by *Ascomycetes*, the fungal partner in the lichen *Cladonia portentosa*, may give an [M-H]^−^ at *m*/*z* 375.1 Da: placodiolic acid (C_19_H_20_O_8_, MW 376.4 g/mol) (**3**), pseudoplacodiolic acid (C_19_H_20_O_8_, MW 376.4 g/mol) (**4**), conprotocetraric acid (C_18_H_16_O_9_, MW 376.3 g/mol) and decarboxythamnolic acid (C_18_H_16_O_9_, MW 376.3 g/mol). Placodiolic and pseudoplacodiolic acids are functional isomeric derivatives of usnic acid (Table 2) and may follow the fragmentation pattern that satisfies tandem MS spectra of peaks eluting at 36.9 min and 37.6 min (Figure 5 and Figure 6) [38,39]. These molecules are tetrahydrodibenzofurans and have been reported in the lichens of genera *Lecanora*, *Rhizoplaca*, *Leprocaulon*, *Phoma* and *Hematoma* [40,41,42]. Interestingly, Shishido et al. have recently reported placodiolic acid in *Cladonia stellaris*, *C. uncialis*, *C. rangiferina*, *C. mitis* and *C. arbuscula* [43], which corroborates its presence in *C. portentosa*. However, mass spectrometry cannot differentiate between placodiolic and pseudoplacodiolic acids [39]. Therefore, it is crucial to isolate and confirm their structures using NMR. The other two lichenic acids corresponding to the chemical formula C_18_H_16_O_9_ and molecular weight 376.3 g/mol include a depsidone, conprotocetraric acid and a depside, decarboxythamnolic acid. These are derivatives of protocetraric and thamnolic acids. respectively, reported in *Cladonia fimbriata*, *C. furcate*, *C. gracilis*, *C. ramulosa*, *C. scabriuscula* and *C. subulate* [20]. However, mass fragmentations of these molecules do not correspond to the MS/MS spectra obtained for the peaks at 36.9 min and 37.6 min. The fifth peak at RT 38.2 min was unambiguously assigned to usnic acid (**5**) based on the fragmentation pattern obtained for the reference standard and corroborated by the literature (Figure 7) [44,45]. The sixth and final peak, eluted at RT 40.3 min with [M-H]^−^ at *m*/*z* 443.2 Da, was determined to be perlatolic acid (**6**, HPLC peak Z) based on the fragmentation pattern and available literature (Figure 8) [45].

The major lichenic acid components were isolated using silica gel column chromatography, followed by further purification through recrystallisation or preparative TLC. The purified compounds were characterised using ESI-MS and NMR. ESI-MS in the negative ion mode showed a single sharp peak at *m*/*z* 223.0979 Da and 237.1133 Da as [M-H]^−^ in the spectra of isolated **1** and **2**, respectively (Supplementary Figures S7 and S8). The ESI-MS spectrum for **5** had a major peak at *m*/*z* 343.0818 Da as [M-H]^−^, and two low intensity peaks at *m*/*z* 709.1503 Da and 1053.2258 Da corresponding to clusters of usnic acid occurring as [2M+Na-2H]^−^ and [3M+Na-2H]^−^, respectively (Supplementary Figure S9). Compound **6** was ionized at *m*/*z* 433.2083 Da as [M-H]^−^, and a fragment ion at 205.1591 Da was obtained through cleavage of the ester bond releasing the 4-*O*-Me-olivetolcarboxylic acid moiety (Supplementary Figure S10).

For NMR, the purified lichenic acids (**1, 5, 6**) were dissolved in deuterated DMSO to conserve protic groups, and compound **2** was dissolved in deuterated chloroform to enable recovery of the sample, which was isolated at a very low yield. ^1^H NMR, ^13^C NMR, HSQC and HMBC were recorded and referenced to solvent. Chemical shifts were assigned based on one-dimensional and two-dimensional NMR (Table 3) and cross-referenced with the literature [34]. HSQC was of less value due to the presence of quaternary carbons, especially in **5**; hence, HMBC was used for thorough structural assignment. The detailed assignment of each molecule showing long-range bond correlation based on HMBC has been provided in the supplementary information (Figures S11–S14). The experimental shift δH values were assigned to all four compounds, and δC for **5** and **2** were in complete agreement with the literature. The δC obtained for compounds **1** and **6** differed slightly from reported values, due to the fact that the NMR spectra of both acids were reported in deuterated acetone in the literature [34]. The δH and δC values assigned to compounds **1** and **6** in the current study were validated using HSQC and HMBC analysis (Supplementary Table S1–S4). The specific rotation of usnic acid was found to be −380 deg [22], indicating a racemic mixture with an enatiomeric excess of 76.7% of (-)-usnic acid. These results are in accordance with the literature [40].

Through bio-guided fractionation, the enzyme inhibitory activity observed in *C. portentosa* extracts has been attributed to **5** and **6**, both of which were reported inhibitors. An assessment of the activity using HPTLC-bioautography was inconclusive due to the suspected false positive behaviour upon visualization. This behaviour has previously been addressed by Taibon et al. in their work on the assessment of tyrosinase enzyme inhibition using this technique [46,47]. HPTLC-bioautography is high-throughput and can be particularly applicable to natural products where complex extracts can be easily separated, allowing dereplication of extract components [47]. It should also facilitate the identification of highly lipophilic leads that are difficult to assess in the aqueous environment of most biological assays. However, compound lipophilicity and poor wettability on the plate is likely to be the cause of false positive behaviour, perhaps also obscuring true positive results. The inclusion of a surfactant in the substrate spray solution, such as Triton X-100, has been shown to improve the result [46].

There is variability in the levels of potency and efficacy reported for **5** in the literature obtained by similar Ellman reaction-based methods using AChE from electric eels. Commercially sourced **5** of unspecified chirality has been reported as an extremely potent nanomolar inhibitor, with an IC_50_ value of 1.273 nM [35]. In sharp contrast, isolated (-)-usnic acid (source unspecified) was found non-active by Studzińska-Sroka et al. while an extract of *Cladonia uncialis* was reported to weakly inhibit the enzyme in the same study [48], a species reported to produce **5** [49]. Another study reported an AChE inhibitory IC_50 value_ of 1.21 µg/mL, which equates to 3.5 µM for **5** isolated from the Antarctic lichen *Himantormia lugubris* [38]. Reddy et al. found that (+)-usnic acid isolated from *Usnea articulata* had negligible inhibition at 100 μM, while in the same study, **6** extracted from *Cladonia portentosa* was found to have an IC_50_ of 6.8 μM [36]. In our case, **5** isolated from *Cladonia portentosa* (−380 deg with 76.7% ee) was a weak inhibitor, providing 25% inhibition at 125 µM, but better than **6** which inhibited the enzyme by approximately 20% at the higher concentration of 250 µM. Under our in vitro conditions, the AchE IC_50_ value of the known inhibitor galanthamine was 2.71 μM. This is comparable to the value reported by Reddy et al., who also used galanthamine as a positive control for inhibition, reporting an IC_50_ value of 2.5 μM, as well as other literature reports [50].

LC–MS analysis revealed six peaks in the hexane extract, four major peaks corresponding to **1**, **2**, **5** and **6** and two minor peaks that may have corresponded to **3** and **4**. Column chromatography and analytical techniques were successful in isolating, purifying and unambiguously characterising the major four lichenic acids, and this is the first report on the isolation of **1** and **2** and identification of **3** and **4** in *Cladonia portentosa*. Compounds **1** and **2** found in lichenic acids have been reported in *Cladonia stellaris* and *Cladonia macaronesica* [34,51,52], while **3** has been reported in *C. stellaris* and other *Cladonia* spp. (discussed earlier) [43]. Lichenic acids such as depsides, dibenzofurans and depsidones are biosynthesised by acetate–polymalonate biosynthetic pathways, starting from phenolic acids and forming orcinolcarboxylic acid intermediaries [53,54]. Compounds **1** and **2** may be biosynthetic precursors that undergo condensation to produce **6**. One may argue that the two monoaryl compounds may be artefacts produced by hydrolysis of the ester bond in **6** during the Soxhlet extraction process, which is performed at elevated temperatures. To test this hypothesis, we utilised three extraction methods to prepare methanol extracts of *C. portentosa*: Soxhlet extraction for 90 min [55], ultrasonication at room temperature for 5 min [56] and mechanical extraction using an IKA homogenizer at room temperature for 5 min. HPLC analysis of the resulting extracts confirmed the extraction of all four lichenic acids in each case. Ultrasonication and mechanical extraction resulted in consistent chemical profiles with little or no degradation, while Soxhlet extraction yielded a higher content of **1** that might indicate some degradation of **6** [unpublished data] due to the longer extraction time and elevated temperature. However, these results validate the presence of the monoaryl compounds **1** and **2** in *C. portentosa*.

## 4. Materials and Methods

### 4.1. Materials

All chemicals including deuterated solvents were purchased from Sigma-Aldrich, Merck unless otherwise stated. Methanol was purchased from Fisher Scientific. Acetylcholinesterase from electric eel, type VI-S, lyophilized power, 374 units/mg solid, was purchased from Merck (Product No. C3389). The enzyme was dissolved in 50 mM Tris-HCl (trisaminomethane hydrochloride) pH 8 buffer and aliquoted at 50 U/50 μL. The aliquots were stored at −80 °C and thawed over ice immediately before use. The ATCI substrate and DTNB were purchased from Fluorochem. Galanthamine hydrobromide, a known acetylcholinesterase inhibitor, was used as a positive control for inhibition, and was purchased from Merck (Product No. 345670).

### 4.2. Methods

#### 4.2.1. Sample Collection and Identification

The lichen sample was collected from an Irish Peatland Conservation Council (IPCC) bog in County Kildare in the mid-east of Ireland (53.270904, −6.939314). The sample was dried at room temperature (RT), exposed to direct sunlight for 24 h and cleaned of debris such as soil, grass, leaves and moss. The sample was identified as *Cladonia portentosa* by lichenologist Dr. Brian Coppins from the Royal Botanic Garden Edinburgh. A voucher sample was prepared and submitted to the National Botanic Gardens of Ireland in Glasnevin, Dublin, and accession code DBN0006287 was obtained.

#### 4.2.2. Soxhlet Extractions

Solvent extractions were carried out through Soxhlet extraction in a Buchi extraction system B-811 [55]. Dried and milled lichen material (2.5 g) was placed in the extraction chamber, and methanol (125 mL) was placed in the solvent beaker attached to the bottom of the apparatus. ‘Soxhlet Standard’ mode was selected and run for 45 min. The extraction process was repeated twice on the material using the same quantities of fresh solvent. The extracts were combined and evaporated to dryness on a rotary evaporator to yield the direct methanol extract. Extracts of the lichen sample were also prepared using a successive extraction process whereby the material was extracted with hexane, followed by ethyl acetate and methanol, yielding three extracts from the sample: a hexane extract, an ethyl acetate successive extract and a methanol successive extract.

#### 4.2.3. HPLC and LC–MS Analysis

A Waters HPLC system consisting of a Waters 600 Controller featuring a quaternary pump and a Waters 717 Plus Autosampler coupled with a Waters 2996 Photo Diode Array (PDA) detector was used for measurements (Milford, MA, USA). The separation was carried out using a 250 × 4.0 mm Grom-Sil 120 ODS-3 CP reverse phase column and a particle size of 5 μm (Dr. Maisch, Ammerbuch, Germany). Chromatographic data for each sample were acquired using a PDA detector from 210 nm to 400 nm, and the chromatograms were initially extracted at 254 nm and 330 nm. A standard HPLC method [37] was applied and then modified to achieve better separation. Linear gradient elution at a flow rate 0.3 mL/min was applied over 60 min in the HPLC starting from 90% of solvent A (H_2_O + 0.1% *v*/*v* formic acid) and 10% of solvent B (acetonitrile solution with 0.1% *v/v* formic acid added) and reaching 10% of solvent A and 90% of solvent B. All the extracts were diluted to a concentration of 100 μg/mL and filtered through 0.45 μm Teflon membrane filters (pre-rinsed with methanol) prior to HPLC analysis. Chromatographic data were acquired and processed using Waters Empower 3 software.

The HPLC-ESI-QTOF-MS/MS platform from Agilent Technologies (Santa Clara, CA, USA) was used to analyze the composition of the extracts. For the separation process, an HPLC chromatograph (1200 series) equipped with a Zorbax Eclipse Plus RP-18 chromatography column (150 mm × 2.1 mm; dp = 3.5 µm), a degasser (G1322A), a binary pump (G1312C), an autosampler (G1329B), a photodiode array detector (G1315D) and a mass spectrometer (G6530B) was used. Agilent MassHunter Workstation Software (version B.10.00, Agilent Technologies, Santa Clara, CA, USA) was used to acquire the MS spectra and process the data. The temperature of the HPLC thermostat was set to 20 °C, and the UV detection wavelengths were set at 210, 230, 254, 280 and 320 nm. The operating wavelength range of the UV-Vis/DAD detector was 190–600 nm. The chromatographic separation was performed using a 10 µL injection volume with a flow rate of 0.2 mL/min, and a 45 min gradient elution program. The mobile phases consisted of eluent A (0.1% formic acid in water, *v*/*v*) and eluent B (acetonitrile solution with 0.1% formic acid, *v*/*v*). The gradient elution was as follows: 0 min—1% of B, 5 min—40% of B, 30 min—55% of B, 31–36 min—95% of B, 37–45 min—1% of B. The post-run duration was set to 10 min. The following settings for the mass spectrometer with electrospray ionisation were applied: gas and sheath gas temperatures of 275 °C and 325 °C, respectively, a gas flow value of 12 L/min, nebulizer pressure of 35 psig, capillary voltage of 3000 V, nozzle voltage of 1000 V, fragmentor voltage of 110 V, skimmer voltage of 65 V, and collision energies of 10 and 20 V. The *m*/*z* range was selected as 40–1200 amu, and the number of precursors per cycle was set to 2 to obtain the most intensive signals. Active exclusion from MS/MS fragmentation of given *m*/*z* signals was introduced for 0.3 min after the collection of 2 spectra. 

#### 4.2.4. Isolation of Lichenic Acids

Column chromatography was performed on silica gel 60 to isolate the components [22]. A total of 300 mg of hexane extract was loaded and eluted with a solvent gradient, starting from neat chloroform and followed by 5%, 10%, 25%, 50% and 75% methanolic chloroform before reaching neat methanol, resulting in 300 fractions. Similar fractions were combined based on their TLC profiles. Compound **5** was eluted with 5% methanolic chloroform, followed by a mixture of **5** and **6** eluted with 10% methanolic chloroform. This was continued with **6** and a mixture of **1** and **2** eluted at 25% and 50% methanolic chloroform, respectively. Compound **5** was purified through crystallization, while the other three were purified using preparative TLC. Column chromatography of the methanol extract was performed in the same fashion to obtain higher amounts of lichenic acids.

#### 4.2.5. Characterisation of Lichenic Acids

Structural investigations were performed using ESI-MS for molecular mass determination and NMR for structural elucidation. ESI mass spectra were acquired using a Bruker micrOTOF-Q III spectrometer interfaced to a Dionex UltiMate 3000 LC in the negative mode. The instrument was calibrated using a tune mix solution (Agilent Technologies ESI-l Low concentration tuning mix), and this was also used as an internal lock mass. The masses were recorded over the range of 100–2000 *m*/*z*. The operating conditions were as follows: endplate offset 500 V, capillary 4500 V, nebulizer 2.0Bar, dry gas 8.0 L/min and dry temperature 180 °C. MicroTof control 3.2 and HyStar 3.2 software were used to carry out the analysis.

A total of 10–15 mg of isolated components were dissolved in 0.75 mL of deuterated DMSO (99.95%) or deuterated chloroform (99.95%) and filtered through prewashed glass wool. ^1^H, ^13^C, HSQC and HMBC NMR data were recorded at 26 °C. ^1^H and ^13^C NMR spectra were generated using a Bruker Avance III 600 NMR spectrometer operating at 600.13 MHz and 150.6 MHz, respectively. All NMR experiments were carried out using pulse programs taken from the Bruker pulse library. The pulse programs used for ^1^H, ^13^C HSQC and HMBC NMR were zg30, zgpg30, hsqcdietgpsisp.2.3 and hmbcetgpl3nd, respectively. In brief, the parameters for ^1^H NMR acquisition were as follows: 32 scans were recorded with an acquisition time of 1.36 s, relaxation delay of 1 s and spectral width of 24,038.5 Hz. For ^13^C NMR acquisition, 32,382 scans were taken with an acquisition time of 0.42 s, relaxation delay of 1 s and spectral width of 39,062.5 Hz. For HSQC NMR acquisition, 2 scans were taken with acquisition time 0.32 s, relaxation delay of 1 s and spectral width of 3238.3, 16,611.3 Hz. For HMBC NMR acquisition, 2 scans were taken with an acquisition time of 0.12 s, relaxation delay of 1 s and spectral width of 8417.5, 31,645.6 Hz. The same parameter settings as ^1^H and ^13^C were employed for two-dimensional HSQC NMR recordings. The spectra were analyzed and processed in MestReNova and TopSpin 3.2 software.

The optical rotations were measured using an AA55 Optical Polarimeter (Optical Activity Ltd., Huntingdon, UK) with a 100 mm cell. Usnic acid solution in chloroform (c = 0.1%) was prepared, and the optical rotation was measured at 25 °C. The specific rotation was calculated using the following formula:

$${\left[\alpha \right]}_{\lambda }^{T}=\frac{\alpha }{c.l}$$

where α = angle of rotation; *l* = polarimeter tube length; *c* = concentration in g/100 mL, T = temperature of solution and λ = wavelength of light source in polarimeter.

In the case of a racemic mixture, enantiomeric excess can be calculated as:

$$enantiomericexcess,ee=\frac{{\left[\alpha \right]}_{observed}}{{\left[\alpha \right]}_{pure}}$$

where [α]_*pure*_ = specific rotation of pure enantiomer and [α]_*observed*_ = specific rotation of racemic mixture.

#### 4.2.6. Anticholinesterase Activity

##### HPTLC-Bioautography

Screening for AChE inhibitory activity was performed using HPTLC-bioautography based on the Ellman method [32]. *C. portentosa* extracts for screening were prepared at a concentration of 1.66 mg/mL in methanol, and volumes within the range 5–15 µL were applied to 20 × 10 cm HPTLC plates (silica gel 60, F_254_, Merck) using the Linomat 5 (CAMAG) under the control of *visionCATS* software version 3.1. The standard application settings were as follows: 15 tracks with 8 mm bands, an 8 mm distance from the lower edge, 11.4 mm between each track and 20 mm from the left and right edges. Galanthamine HBr 5 mM in methanol was included on the plates as a positive control for enzyme inhibition. The plates were developed using an automatic development chamber (ADC2) (CAMAG) with standard settings: 30 s pre-drying, 10 min chamber saturation with filter paper and 5 min of plate drying after development. The mobile phase used was CHCl_3_:MeOH:H_2_O (75:23:2). The developed and dry plates were visualized under a UV light at 254 nm. They were then placed in a Derivatizer (CAMAG) and sprayed with 4 mL of a solution consisting of 2 mM ATCI and 2 mM DTNB in 50 mM Tris-HCl buffer, pH 8 (Buffer A) until saturated. The plates were allowed to dry for 4 min and then sprayed with 4 mL of AChE enzyme at a concentration of 5 U/mL in Buffer A. After 10–15 min, the appearance of each plate was recorded using a TLC Visualizer (CAMAG) with white light in remission and with white light in transmission.

##### Colorimetric Microplate Assay for AChE Inhibition

AChE inhibitory activities were quantitatively examined using a colorimetric microplate assay based on the Ellman method with some modifications, as described by Rhee et al. [31]. The AChE enzyme was prepared at 0.25 U/mL in 50 mM Tris-HCl pH 8 containing 0.1% w/v of bovine serum albumin. Each test well in triplicate contained 45 µL of the prepared enzyme solution, 125 µL of 3 mM DTNB in 50 mM Tris-HCl pH 8 containing 0.1 M NaCl and 0.02 M MgCl_2_.6H_2_O, the sample and Tris HCl pH 8 buffer to a well volume of 225 μL. The following controls were included in triplicate on the plate: C1 (no inhibitor control) contained buffer in place of sample, C2 (no enzyme control) contained enzyme dilution buffer in place of the prepared enzyme solution and buffer in place of sample and C3 (solvent control) contained the inhibitor candidate vehicle in place of the sample. *C. portentosa* extracts were prepared in methanol for the assay, and the maximum final well concentration of methanol was limited to 10%. Isolated lichenic acid components were prepared in DMSO, and the DMSO concentration was limited to 0.5%. The plate was incubated for 15 min at RT, then the reaction was initiated by the addition of 25 μL of 15 mM ATCI in H_2_O.

The absorbance was measured at 405 nm at time t= 0 and t = 10 min using a FLUOstar OPTIMA microplate reader (BMG LabTech). Acetylcholinesterase inhibition was calculated as:

$$\%\;\mathrm{Inhibition}=1-\frac{\Delta A_{Test}}{\Delta A_{Solventcontrol}}\times 100$$

where:

∆A_Test_ = absorbance of a test well at t = 10 min minus absorbance at t = 0 min
∆A_Solvent control_ = average absorbance of C3 (solvent control) wells at t = 10 min minus absorbance at t = 0 min

Enzyme inhibition curves were prepared, and statistical analyses were performed in GraphPad^®^ Prism 5.01 for Windows.

## 5. Conclusions

In the delineation of the AChE inhibitory effects of *Cladonia portentosa*, four lichenic acids—olivetolic, 4-*O*-Me-olivetolcarboxylic, usnic and perlatolic acids—were isolated and characterised. This is the first report on the isolation of olivetolic and 4-*O*-Me-olivetolcarboxylic acids and identification of placodiolic and pseudoplacodiolic acids from the lichen *C. portentosa*. The anticholinesterase activity of its extract can be attributed to the usnic acid and perlatolic acid components. As usnic and perlatolic acids have been known for cytotoxicity [21], in-house studies are being conducted to evaluate the cytotoxicity and anti-inflammatory activities of the two new compounds—olivetolic and 4-O-methylolivetolcarboxylic acids.

## Figures and Tables

**Table 1:** Extract yields.

S. No.	Extract	Direct/Successive	Yield (%)
1	Methanol	Direct, Soxhlet	14.40
2	Hexane	Successive, Soxhlet	1.36
3	Ethyl acetate	Successive, Soxhlet	0.98
4	Methanol	Successive, Soxhlet	7.56

**Table 2:** Tentatively identified components of *Cladonia portentosa* hexane extract in the HPLC-ESI-QTOF-MS/MS-based negative ion experiment; RT: retention time; DBE: double bond equivalent.

No	Ion.+/-	RT [min]	Proposed Structure	Molecular Formula	*m*/*z* Theoretical	*m*/*z* Experimental	Error	DBE	MS/MS Fragmentation	Proposed Compound
1	-	17.6	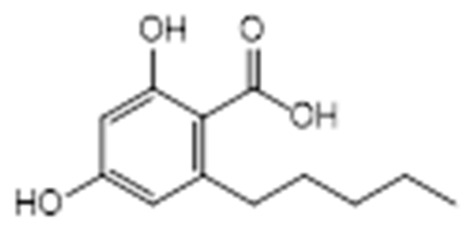	C_12_H_16_O_4_	223.0976	223.0997	−9.45	5	205, 179, 137	Olivetolic acid (**1**)
2	-	29.9	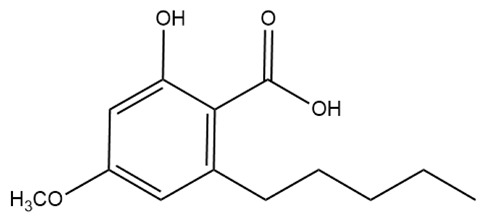	C_13_H_18_O_4_	237.1132	237.1158	−10.78	5	193, 178, 149, 122	4-O-methylolivetolcarboxylic acid (**2**)
3,4	-	36.9 and 37.6	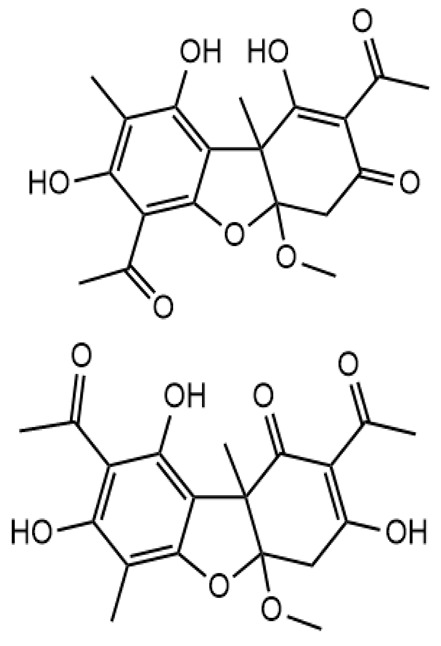	C_19_H_20_O_8_	375.1085	375.1134	−10.26	10	343, 299, 259, 180, 139	Placodiolic acid (**3**) or Pseudoplacodiolic acid (**4**)
5	-	38.2	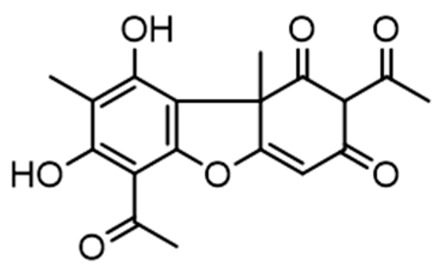	C_18_H_16_O_7_	343.0955	343.0862	−11.3	11	328, 299, 259, 231	Usnic acid (**5**)
6	-	40.3	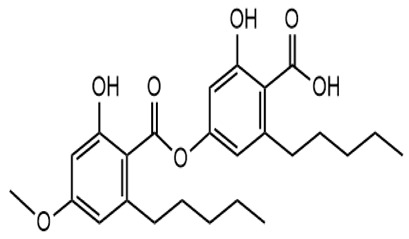	C_25_H_32_O_7_	443.2075	443.2109	−7.59	10	399, 355, 223, 205, 179	Perlatolic acid (**6**)

## Data Availability

Research data on the characterization of isolated molecules has been provided in the supplementary data.

## Supplementary Materials

The following supporting information can be downloaded at: https://www.mdpi.com/article/10.3390/molecules28104145/s1, Figure S1: MS/MS spectrum corresponding to the peak X at RT=17.6 min in LC–MS chromatogram tentatively identified as olivetolic acid; Figure S2: MS/MS spectrum corresponding to the peak Y at RT=29.9 min in LC–MS chromatogram tentatively identified as 4-O-methylolivetolcarboxylic acid; Figure S3: MS/MS spectrum corresponding to the peak at RT=36.9 min in LC–MS chromatogram tentatively identified as pseudoplacodiolic acid or placodiolic acid; Figure S4: MS/MS spectrum corresponding to the peak at RT=37.6 min in LC–MS chromatogram tentatively identified as pseudoplacodiolic acid or placodiolic acid; Figure S5: MS/MS spectrum corresponding to the peak at RT= 38.2 min in LC–MS chromatogram tentatively identified as usnic acid; Figure S6: MS/MS spectrum corresponding to the peak Z at RT= 40.3 min in LC-MS chromatogram tentatively identified as perlatolic or perlatolinic acid; Figure S7: ESI-MS spectrum of isolated olivetolic acid in negative ion mode; Figure S8: ESI-MS spectrum of isolated 4-*O*-methylolivetolcarboxylic acid in negative ion mode; Figure S9: ESI-MS spectrum of isolated usnic acid in negative ion mode; Figure S10: ESI-MS spectrum of isolated perlatolic or perlatolinic acid in negative ion mode; Figure S11: NMR spectra of isolated olivetolic acid; Table S1: HMBC based assignment of olivetolic acid; Figure S12: NMR spectra of isolated 4-*O*-methylolivetolcarboxylic acid; Table S2: HMBC-based assignment of 4-*O*-methylolivetolcarboxylic acid; Figure S13: NMR spectra of isolated usnic acid; Table S3: HMBC-based assignment of usnic acid; Figure S14: NMR spectra of isolated perlatolic acid; Table S4: HMBC-based assignment of perlatolic acid.
