# Supplementary material for: Unravelling Novel Phytochemicals and Anticholinesterase Activity in Irish Cladonia portentosa
Source: Molecules. 2023 May 17;28(10):4145. doi: 10.3390/molecules28104145 (PMC10222842; doi:10.3390/molecules28104145)
Supplement: Supplementary file 1 [file molecules-28-04145-s001.zip › molecules-2361997-supplementary.pdf]

Unravelling novel phytochemicals and anti-cholinesterase activity in Irish *Cladonia portentosa*

Shipra Nagar [1,*], Maria Pigott [1], Wirginia Kukuła-Koch [2] and Helen Sheridan [1,*]

[1] NatPro Centre, School of Pharmacy and Pharmaceutical Sciences, Trinity College Dublin, Dublin 02, D02 PN40 Dublin, Ireland
[2] Department of Pharmacognosy, Medical University of Lublin, 1 Chodzki street, 20-093 Lublin, Poland
* Correspondence: nagars@tcd.ie or shhipranagar@gmail.com (S.N.); hsheridn@tcd.ie (H.S.)

Supplementary Information

Appendix A: MS/MS spectra of LCMS Chromatogram (figure 2)

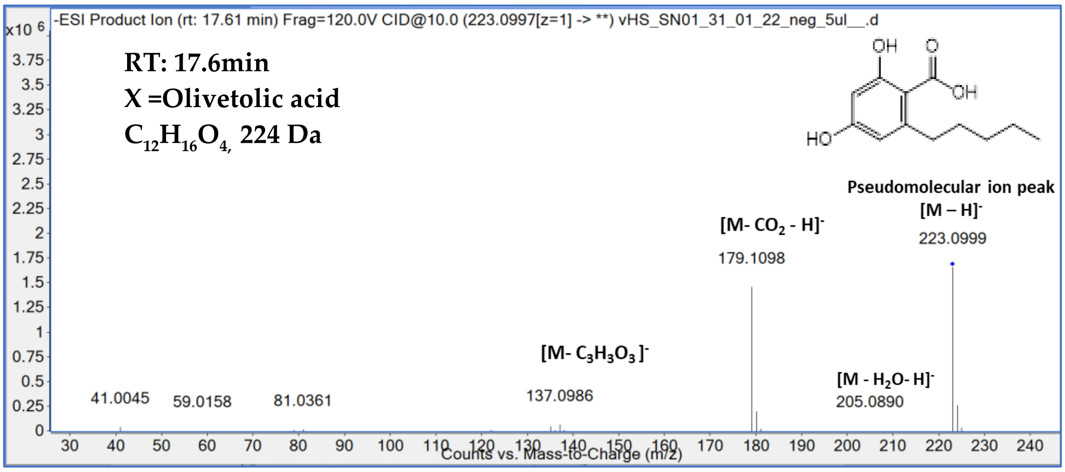

Figure S1: MS/MS spectrum corresponding to the peak X at RT=17.6 min in LC-MS chromatogram (Figure 2 in manuscript), tentatively identified as olivetolic acid

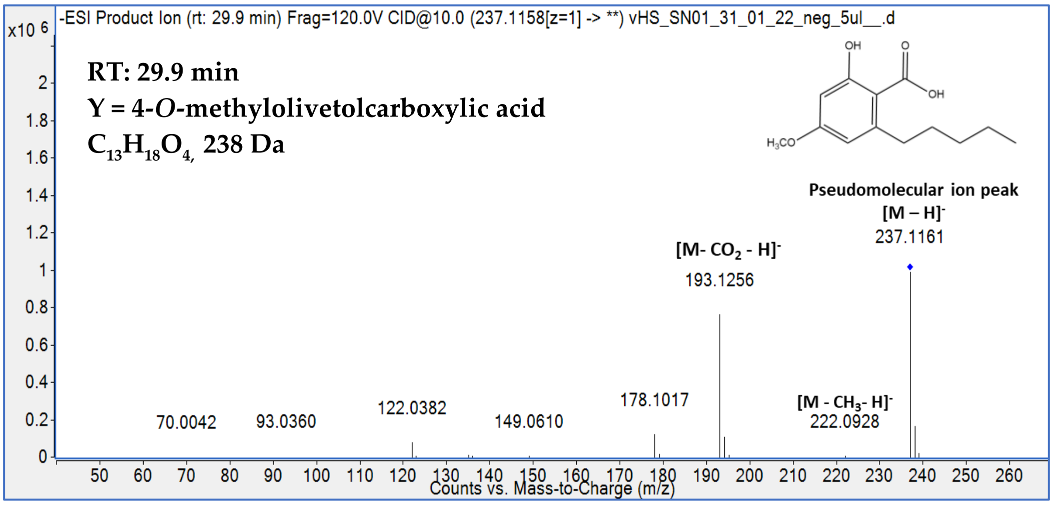

Figure S2: MS/MS spectrum corresponding to the peak Y at RT=29.9 min in LC-MS chromatogram (Figure 2 in manuscript), tentatively identified as 4-*O*-methylolivetolcarboxylic acid

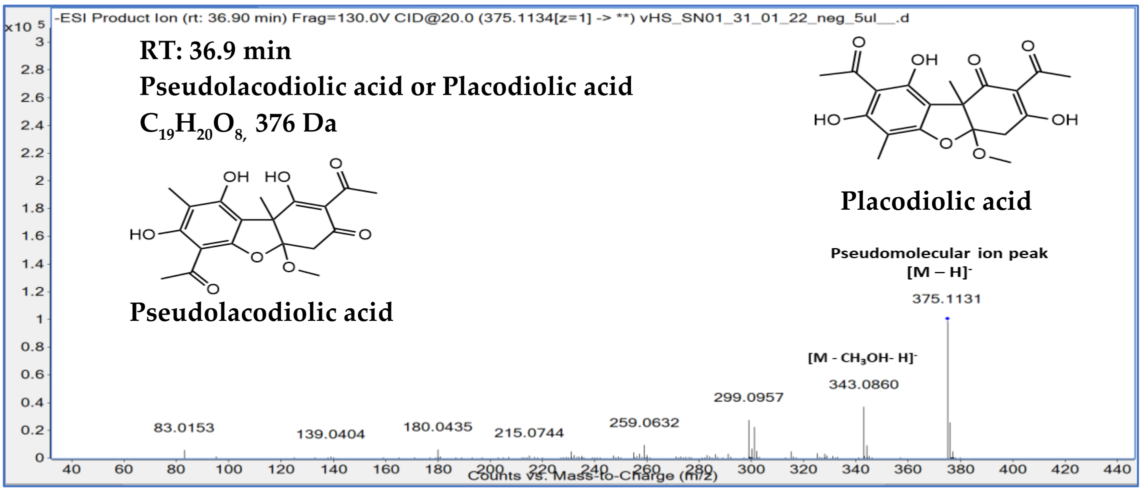

Figure S3: MS/MS spectrum corresponding to the peak at RT=36.9 min in LC-MS chromatogram (Figure 2 in manuscript), tentatively identified as pseudoplacodiolic acid or placodiolic acid

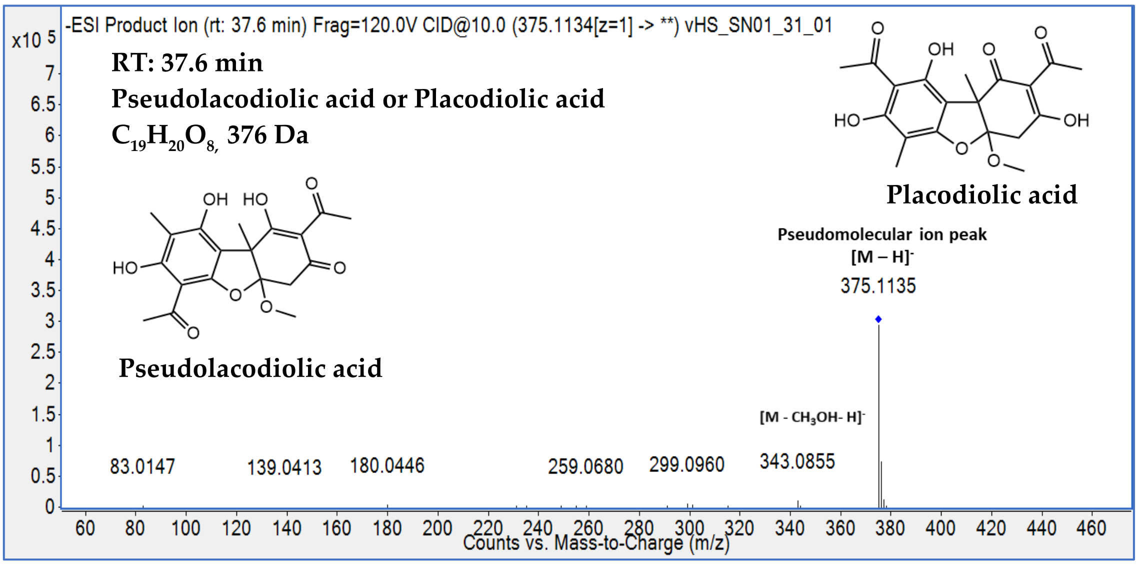

Figure S4: MS/MS spectrum corresponding to the peak at RT=37.6 min in LC-MS chromatogram (Figure 2 in manuscript), tentatively identified as pseudoplacodiolic acid or placodiolic acid

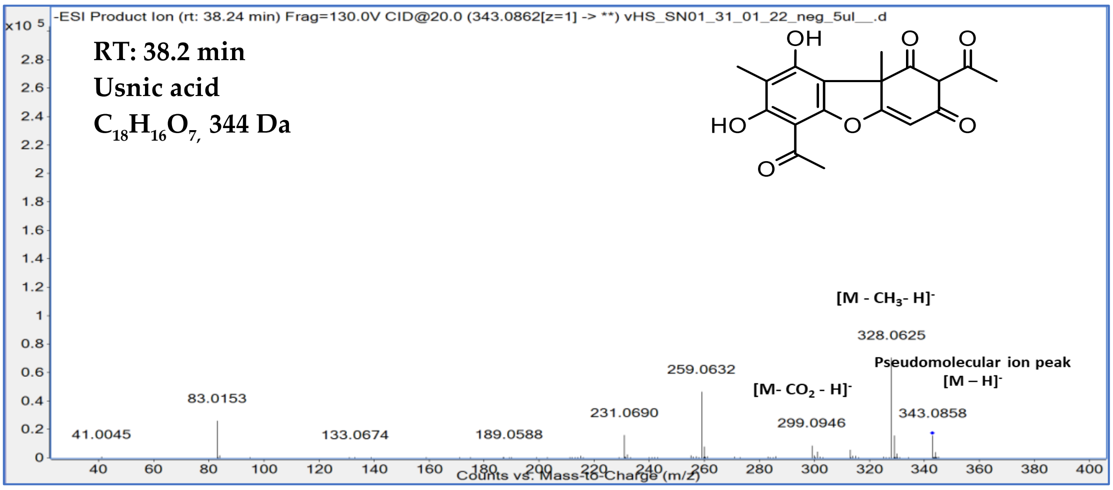

Figure S5: MS/MS spectrum corresponding to the peak at RT= 38.2 min in LC-MS chromatogram (Figure 2 in manuscript), tentatively identified as usnic acid

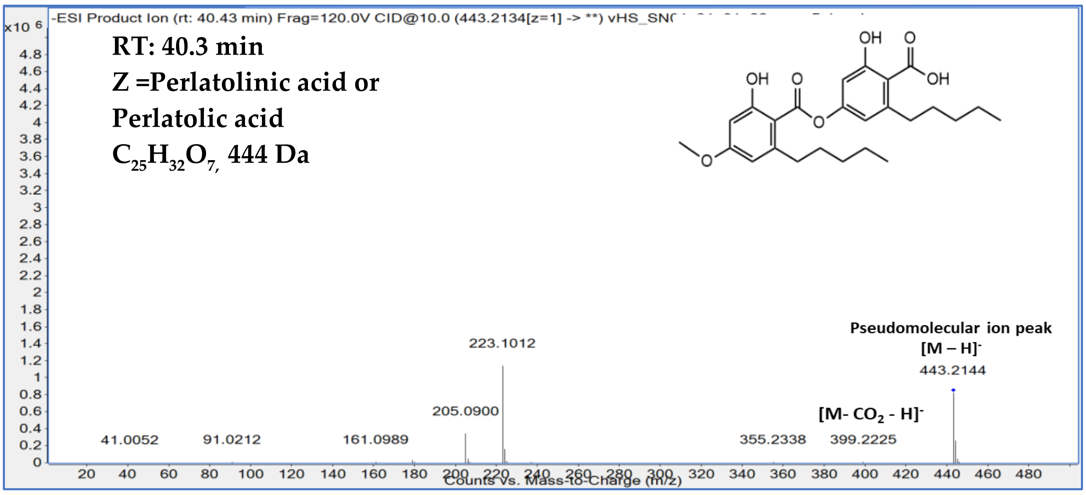

Figure S6: MS/MS spectrum corresponding to the peak Z at RT= 40.3 min in LC-MS chromatogram (Figure 2 in manuscript), tentatively identified as perlatolic or perlatolinic acid

Appendix B: ESI-MS spectra of Isolated Compounds

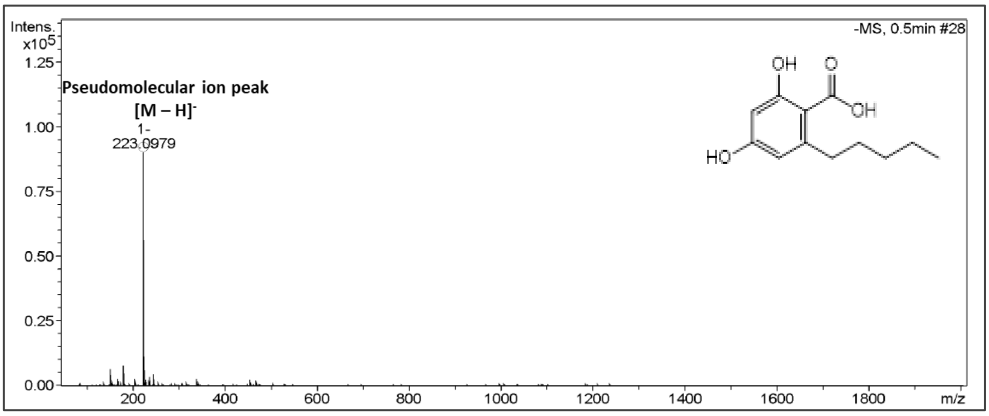

Figure S7: ESI-MS spectrum of isolated olivetolic acid in negative ion mode

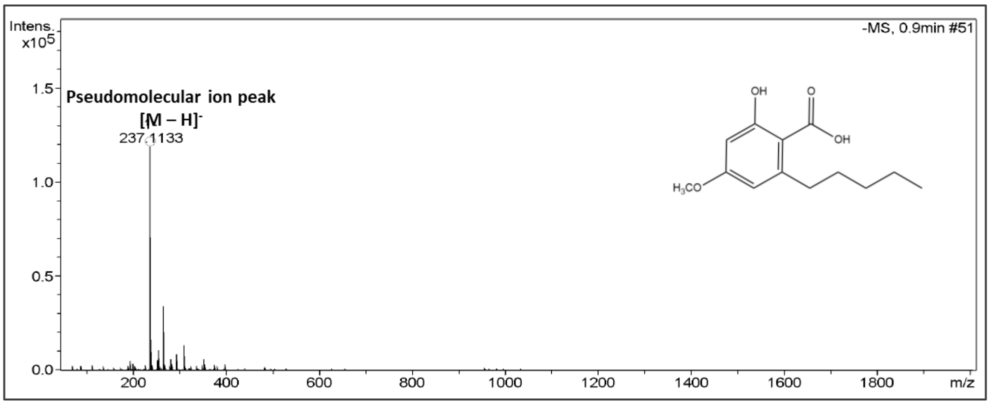

Figure S8: ESI-MS spectrum of isolated 4-*O*-methylolivetolcarboxylic acid in negative ion mode

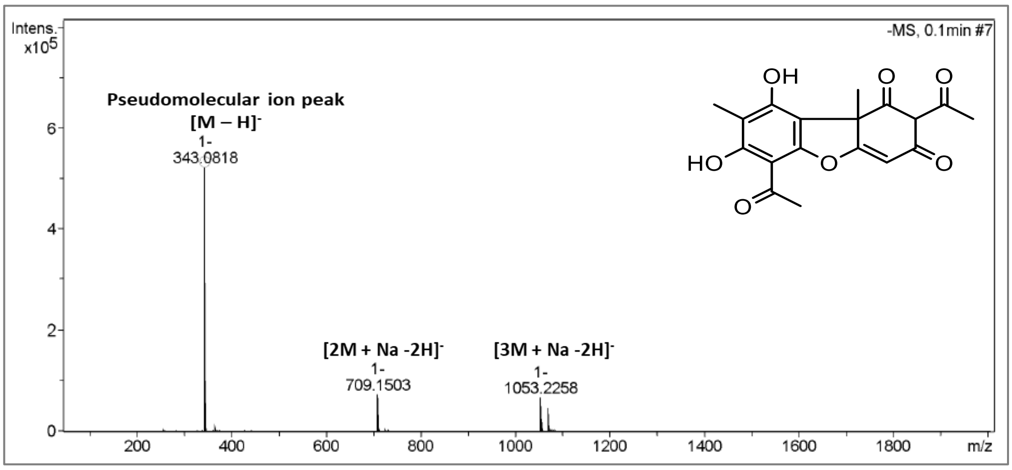

Figure S9: ESI-MS spectrum of isolated usnic acid in negative ion mode

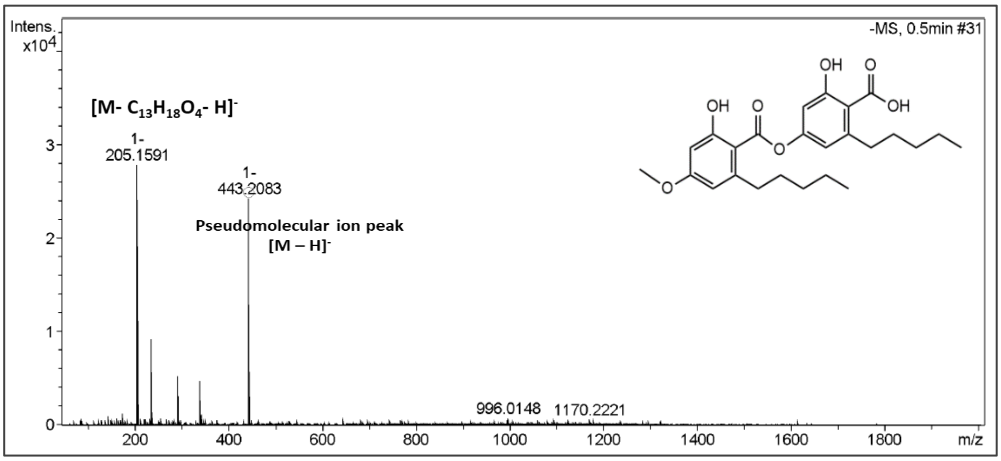

Figure S10: ESI-MS spectrum of isolated perlatolic or perlatolinic acid in negative ion mode

Appendix C: NMR Assignment of Isolated Compounds

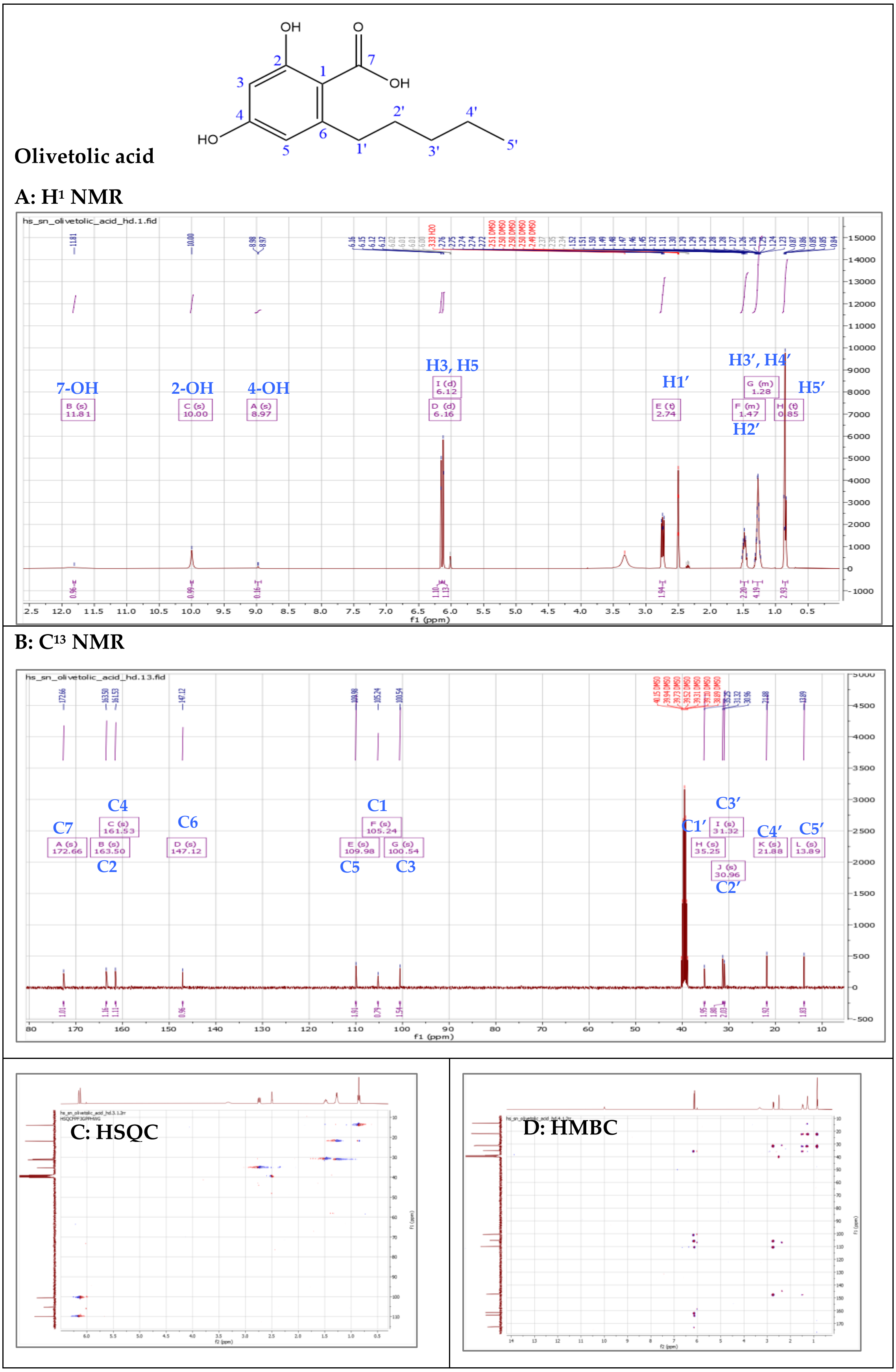

Figure S11: NMR spectra of isolated olivetolic acid

Table S1: HMBC based assignment of olivetolic acid

Position	δH (ppm)	δC (ppm)	HMBC correlations
1	-	105.2	
2-OH	10.00 (s)	163.5	
3	6.12 (d)	100.5	163.5 (C2), 161.5 (C4), 105.2 (C1), 109.9 (C5)
4	-	-	
4-OH	8.97 (s)	161.5	
5	6.16 (d)	109.9	163.5 (C2), 161.5 (C4), 105.2 (C1), 100.5 (C3), 35.2 (C1’)
6	-	147.1	
7	-	172.7	
7-OH	11.81 (s)	-	
8	-	-	
8-OH	-	-	
9-OH	-	-	
1’	2.74 (t)	35.2	31.3 (C3’), 31.0 (C2’), 109.9 (C5), 147.1 (C6), 105.2 (C1)
2’	1.47 (m)	30.9	147.1 (C6), 35.2 (C1’), 31.3 (C3’), 21.8 (C4’)
3’	1.28 (m)	31.3	21.8 (C4’), 13.9 (C1’)
4’	1.28 (m)	21.8	31.3 (C3’), 13.9 (C1’)
5’	0.85 (t)	13.9	31.3 (C3’), 21.8 (C4’)

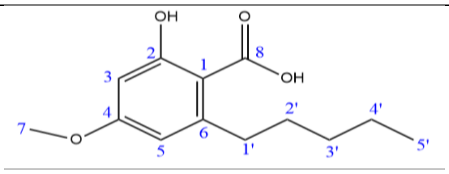

4-*O*-methylolivetolcarboxylic acid

A: H^1 NMR

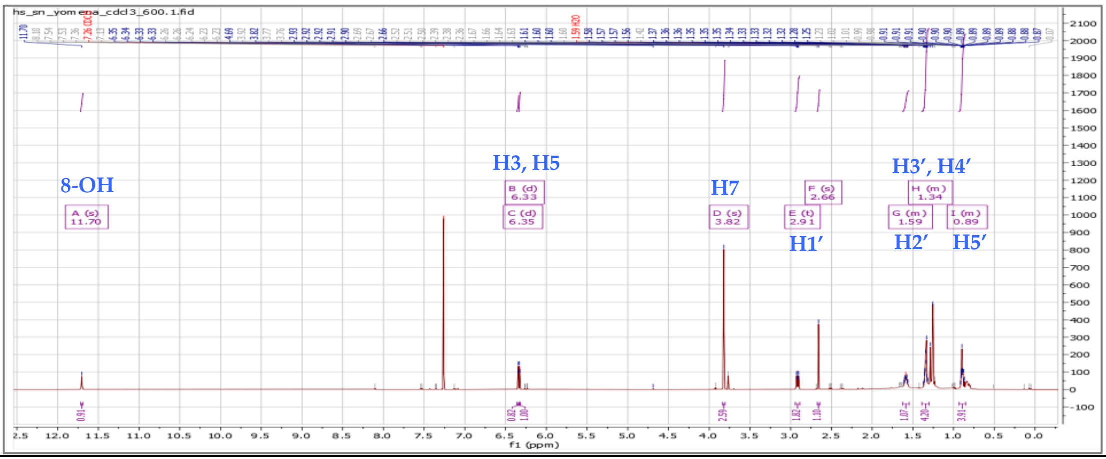

B: C^{13} NMR

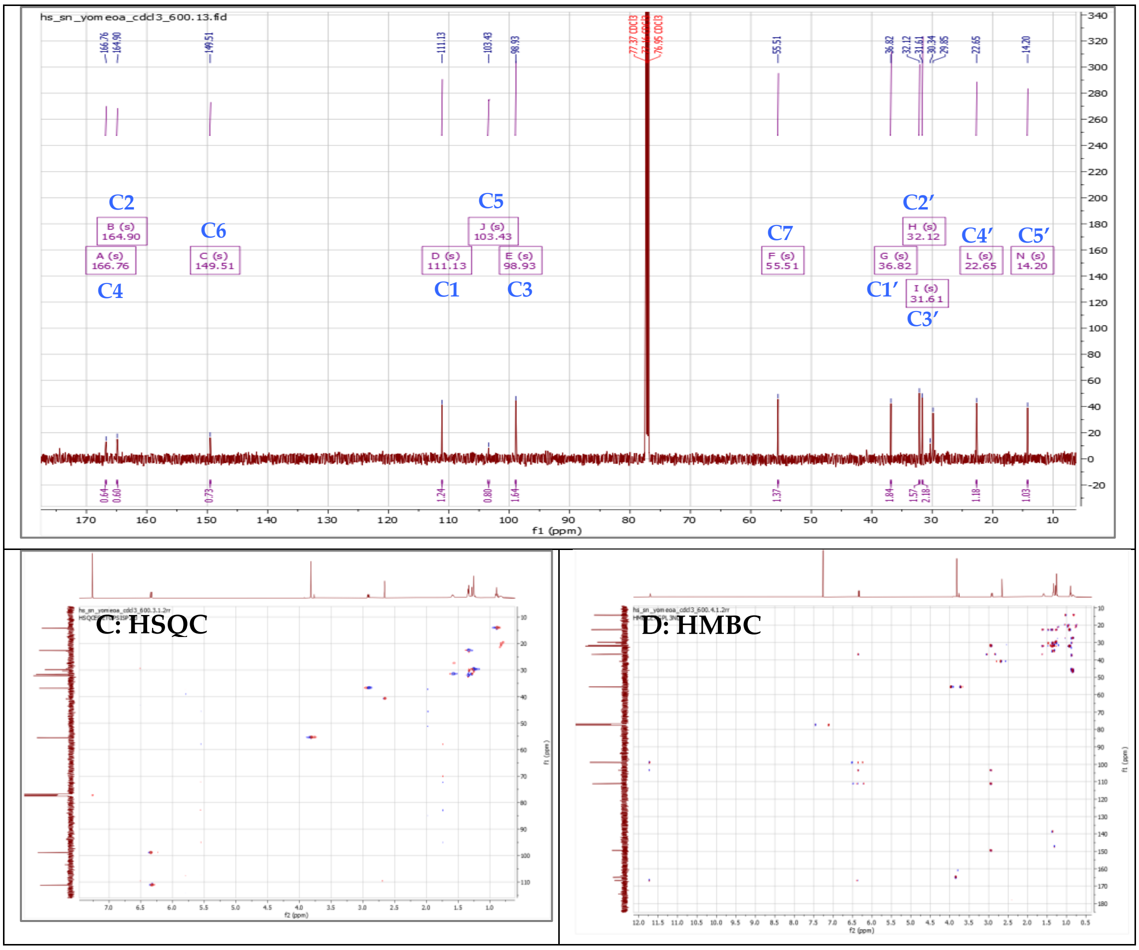

Figure S12: NMR spectra of isolated 4-*O*-methylolivetolcarboxylic acid

Table S2: HMBC based assignment of 4-*O*-methylolivetolcarboxylic acid

Position	δH (ppm)	δC (ppm)	HMBC correlations
1	-	111.3	
2-OH	-	164.9	
3	6.33 (d)	98.9	103.4 (C5)
4	-	166.8	
4-OH	-	-	
5	6.35 (d)	103.4	36.8 (C1’), 103.4 (C5), 98.9 (C3), 166.8 (C4)
6	-	149.5	
7	3.82 (s)	55.5	164.9 (C2)
7-OH	-	-	
8	-	-	
8-OH	11.70 (s)	-	98.9 (C3), 103.4 (C5)
9-OH	-	-	
1’	2.91 (t)	36.8	103.4 (C5), 111.3 (C1), 149.5 (C6), 31.6 (C3’)
2’	1.59 (m)	32.1	36.8 (C1’), 22.6 (C4’)
3’	1.34 (m)	31.6	149.5 (C6), 32.1 (C2’), 22.6 (C4’)
4’	1.34 (m)	22.6	32.1 (C2’)
5’	0.89 (m)	14.2	36.8 (C1’), 32.1 (C2’), 22.6 (C4’)

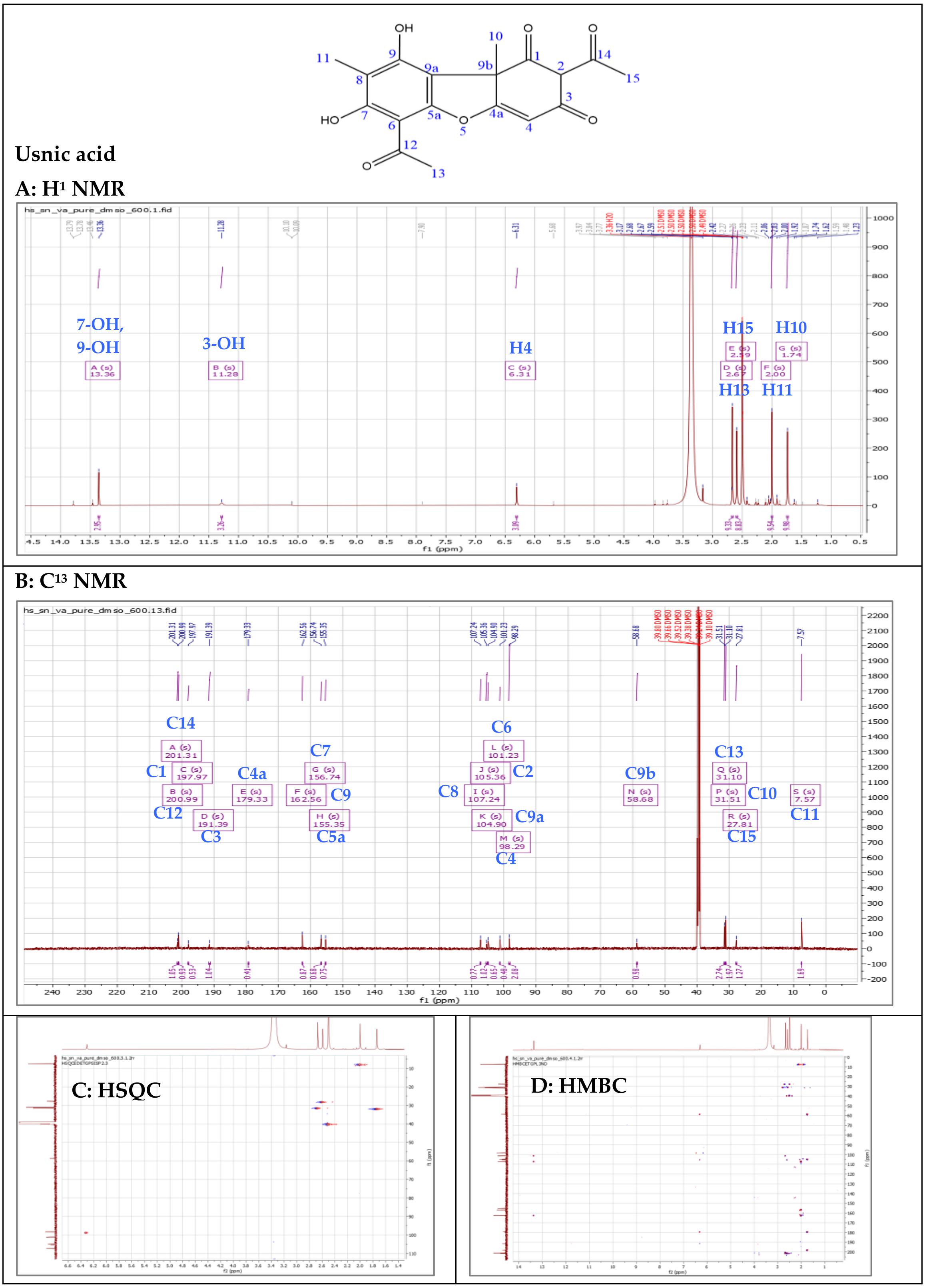

Figure S13: NMR spectra of isolated usnic acid

Table S3: HMBC based assignment of usnic acid

Position	δH (ppm)	δC (ppm)	HMBC correlations

1	-	197.9	
2	-	105.4	
3	-	191.4	
3-OH	11.28 (s)		
4	6.31 (s)	98.3	58.7 (C9b), 105.4 (C2), 179.3 (C4a), 191.4 (C3)
4a		179.3	
5	-	-	
5a		155.4	
6	-	101.2	
7	-	156.7	
7-OH	13.36 (s)	-	162.6 (C9), 107.2 (C8), 101.2 (C6)
8	-	107.2	
9		162.6	
9-OH	13.36 (s)		162.6 (C9), 107.2 (C8)
9a	-	104.9	
9b	-	58.7	
10	1.74 (s)	31.5	104.9 (C9a), 58.7 (C9b), 179.3 (C4a), 197.9 (C1)
11	2.00 (s)	7.6	156.7 (C7), 162.6 (C9)
12	-	201.0	
13	2.67 (s)	31.1	201.0 (C12)
14	-	201.3	
15	2.59 (s)	27.8	201.3 (C14)

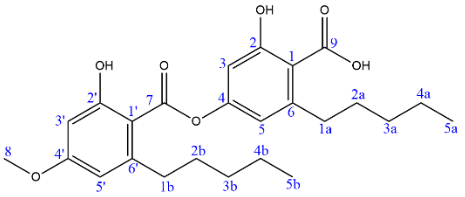

Perlatolic acid

A: H^1 NMR

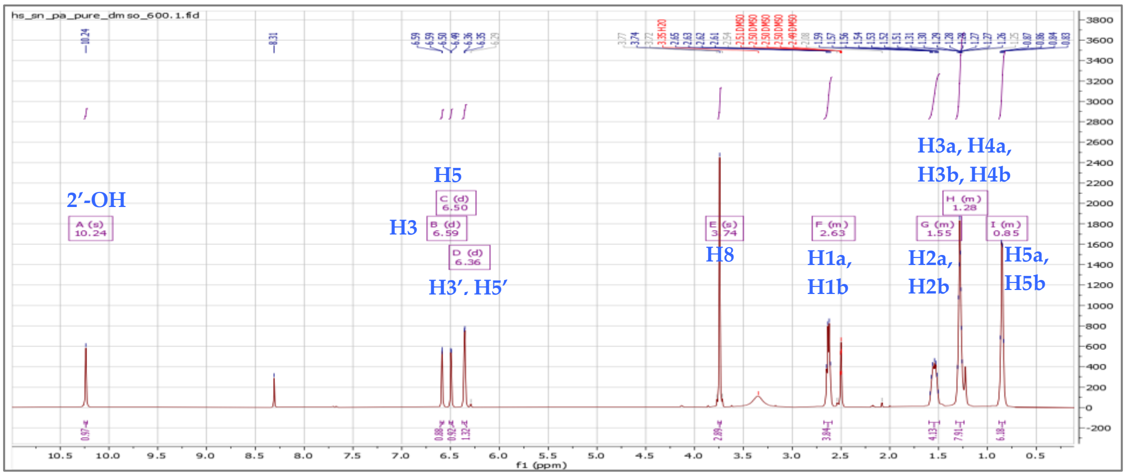

B: C^{13} NMR

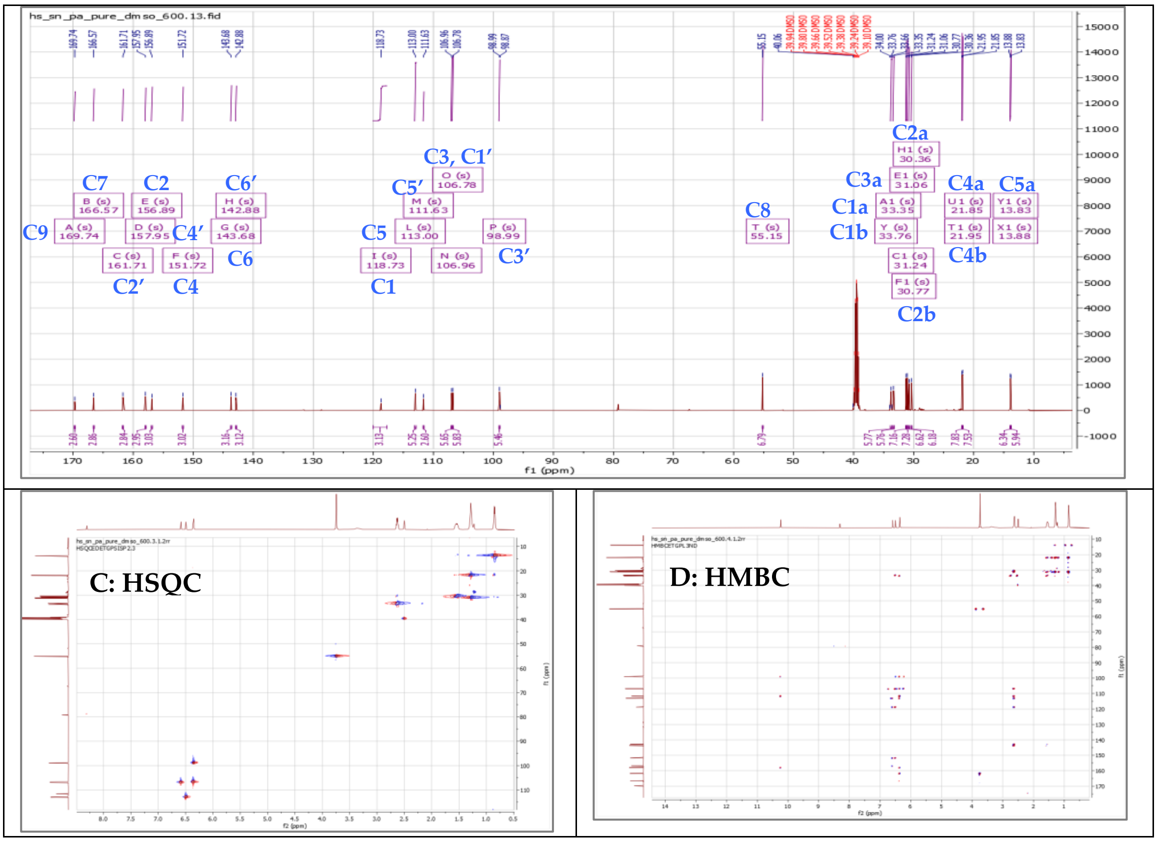

Figure S14: NMR spectra of isolated perlatolic acid

Table S4: HMBC based assignment of perlatolic acid

Position	δH (ppm)	δC (ppm)	HMBC correlations
**1**	-	118.7	
**2-OH**	-	156.9	
**3**	6.59 (d)	106.8	156.9(C2), 151.7 (C4), 113.0 (C5)
**4**	-	151.7	
**4-OH**	-	-	
**5**	6.50 (d)	113.0	106.8(C3), 151.7 (C4), 33.3 (C1a),
**6**	-	143.7	
**7**	-	166.6	
**7-OH**	-	-	
**8**	3.74 (s)	55.2	161.7 (C2’)
**8-OH**	-	-	
**9-OH**	-	169.7	
**1’**	-	106.8	
**2’-OH**	10.24 (s)	161.7	111.6 (C5’), 157.9 (C4’)
**3’**	6.36 (d)	98.9	106.8(C1’), 161.7(C2’), 157.9 (C4’), 111.6(C5’)
**4’**	-	157.9	
**5’**	6.36 (d)	111.6	106.8(C1’), 98.9(C3’), 157.9 (C4’), 33.8 (C1b)
**6’**	-	142.9	
**1a**	2.63 (m)	33.3	118.7 (C1), 113.0 (C5), 143.7(C6), 30.4 (C2a), 31.1 (C3a)

**2a**	1.55 (m)	30.4	33.3 (C1a), 31.1 (C3a)
**3a**	1.28 (m)	31.1	21.8(C4a), 13.8 (C5a)
**4a**	1.28 (m)	21.8	31.1 (C3a), 13.8 (C5a)
**5a**	0.85 (t)	13.8	21.8 (C4a), 31.1(C3a), 30.4(C2a)
**1b**	2.63 (m)	33.8	106.8(C1’), 111.6(C5’), 142.9(C6’), 30.8 (C2b),
**2b**	1.55 (m)	30.8	33.8 (C1b), 31.2 (C3b)
**3b**	1.28 (m)	31.2	21.9 (C4b), 13.9 (C5b)
**4b**	1.28 (m)	21.9	13.9 (C5b)
**5b**	0.85 (m)	13.9	21.9 (C4b), 31.2 (C3b), 30.8 (C2b)
